# Alterations of placental HERV expression as a result of gestational COVID-19 disease

**DOI:** 10.3389/fmicb.2026.1901506

**Published:** 2026-09-11

**Authors:** Ana Medel-Martinez, Mattia Bugatti, Mariana Polychronopoulou, Mark Strunk, Sofía Macías-Redondo, Andrea Bazo, Marta Garrido-Pontnou, Konstantina Kitsou, Marta Fabre, Cristina Paules, Daniel Oros, Justine Pierquin, Joanna Brunel, Benjamin Charvet, Hervé Perron, Fabio Facchetti, Gkikas Magiorkinis, Jon Schoorlemmer

**Affiliations:** 1 Instituto de Investigación Sanitaria de Aragón (IIS Aragón), Zaragoza, Spain; 2 Maternal and Fetal Health Research Group, GIIS-106, IISA, Zaragoza, Spain; 3 Instituto Aragonés de Ciencias de la Salud (IACS), Centro de Investigación Biomédica de Aragón (CIBA), Zaragoza, Spain; 4 Pathology Unit, Department of Molecular and Translational Medicine, University of Brescia, Brescia, Italy; 5 Department of Preventive Medicine, Epidemiology and Medical Statistics, Medical School, National and Kapodistrian University of Athens, Athens, Greece; 6 Pathology Department, Hospital Universitari Vall d’Hebron, Barcelona, Spain; 7 Placental Pathophysiology & Fetal Programming Research Group, GIIS-028, IISA, Zaragoza, Spain; 8 Biochemistry Department, Hospital Clínico Universitario Lozano Blesa, Zaragoza, Spain; 9 Obstetrics Department, Hospital Clínico Universitario Lozano Blesa, Zaragoza, Spain; 10 Red de SaludMaterno Infantil y del Desarrollo (SAMID), RETICS, Instituto de Salud Carlos III (ISCIII), Subdirección General de Evaluación y Fomento de la Investigación y Fondo Europeo de Desarrollo Regional (FEDER), Madrid, Spain; 11 University of Zaragoza, Zaragoza, Spain; 12 GeNeuro Innovation, Lyon, France; 13 ARAID Foundation, Zaragoza, Spain

**Keywords:** COVID-19, HERVW ENV, human endogenous retroviruses, placenta, pregnancy, placental damage, adverse pregnancy outcomes (APOs)

## Abstract

**Introduction:**

Activation of human endogenous retroviral elements (HERV) is a well-documented phenomenon in inflammatory pathologies including COVID-19, with HERVW ENV frequently mentioned as a toxic protein. Given that COVID-19 during pregnancy has been associated with placental damage and a higher risk of adverse pregnancy outcomes (APOs), we investigated whether gestational COVID-19 alters placental HERV expression, and if such alterations correlate with placental damage or dysfunction.

**Methodology and results:**

We quantitively analyzed mRNA expression of HERV in placental samples using qPCR assays rigorously tested for efficiency and specificity or transcriptomic analysis. Combined qPCR and transcriptomic analyses of placentas from maternal COVID-19 cases revealed an overall trend toward reduced expression of several HERV elements, including HERVW ENV-related transcripts, with a more pronounced downregulation in SARS-CoV-2-positive placentas. HERVW ENV protein was detected by immunofluorescence in the syncytiotrophoblast (STB), co-staining with HCGβ, using conditions minimizing cross-reactivity with ERVWE1. HERVW ENV protein levels were diminished in COVID-19 patients, especially in those cases in which SARS-CoV-2 was present in the placenta.

**Conclusions and discussion:**

While HERV expression is reportedly induced in blood lymphocytes and other tissues as a response to SARS-CoV-2 infection, we detect unaltered or lower RNA levels of selected retroviral elements in the placenta of COVID-19 patients. HERVW ENV protein was present in non-COVID-19 placenta, and downregulated by maternal COVID-19 disease. The absence of HERVW ENV protein induction was confirmed by the careful study of cases with placental SARS-CoV-2 infection, as HERVW ENV was mostly absent from these samples, probably as an indirect result of placental damage. We identified two independent triggers for the downregulation of both HERVW ENV RNA and protein in placenta: maternal COVID-19 and local effects of SARS-CoV-2. Therefore, this HERV downregulation may represent part of the molecular response to placental SARS-CoV-2 infection, contributing to placental dysfunction and APOs.

## 1 Introduction

Retrotransposons are genetic elements that replicate through an RNA intermediate and reintegrate into the genome, playing a significant role in genomic structure and evolution (Kazazian, 2004). Among them, human endogenous retroviruses (HERVs) are a specific class of retrotransposons that contain long terminal repeats (LTRs), distinguishing them from non-LTR retrotransposons such as LINE-1 (Long Interspersed Nuclear Element-1; Bannert and Kurth, 2004). HERVs account for approximately 8% of the human genome and are classified into 31 families, each originating from an independent ancestral retroviral infection (Vargiu et al., 2016). These elements arise when a retroviral genome integrates into the germline DNA as a provirus—a form in which the viral genes and two identical LTRs become a permanent part of the host genome (Johnson, 2019). Most of the HERVs copies do not contain the full-length proviral sequence due to the recombination events that occur between the two LTRs of the original proviral insertion (Thomas et al., 2018). As a result, in addition to full length provirus, three subsets of transcriptionally incompetent copies (pseudoelements, truncated copies and solo LTR copies; Costas, 2002; Li et al., 2011) are distributed throughout the genome.

HERVs are grouped into several families based on sequence homology including HERVW, HERVFRD, HERVE, HERVH, HERVI, HERVL and HERVK. Although the vast majority of HERVs is not actively transcribed nor translated into proteins under biological circumstances (Oja et al., 2007), it is widely documented that the expression of specific HERVs is involved in physiological (Da Silva et al., 2024) and pathological processes (Ko and Cha, 2021), both as non-coding RNAs or translated proteins. This is especially true for placenta. Envelope (ENV) proteins from both HERVFRD and HERVW families have been related to placental development and fetal-maternal immune tolerance (Mangeney et al., 2007; Lavialle et al., 2013; Grandi and Tramontano, 2018; Da Silva et al., 2024). The best-known examples are the human syncytin-1/*ERVWE1* and syncytin-2/*ERVFRD1* which are products of envelope genes of defective human endogenous retroviruses of the HERVW and HERVFRD families, respectively. Both ERVWE1 and ERVFRD1 work cooperatively to ensure proper physiological function, with syncytin-1 primarily facilitating cellular fusion processes (Mi et al., 2000) and syncytin-2 contributing to both cell fusion and immunomodulation (Vargas et al., 2009). Apart from syncytin proteins, HERVH, who is known to play a crucial role in embryonic development (Carter et al., 2022), has been recently associated to the regulation of trophoblast differentiation/function through the expression of the HERVH derived-long non-coding RNA (lncRNA) *UCA1* (Kong et al., 2024). The K clade of HERVs is closely related to the exogenous mouse mammary tumor virus (MMTV; Garcia-Montojo et al., 2018). HERVK consists of 10 groups, namely HML-1 to −10, that were classified based on the reverse transcription (RT) gene sequence. The most recently active HERV family is HERVK HML-2, which can be found dispersed throughout our genome in both proviral and solo LTR form, while carrying intact ORFs for some genes (Subramanian et al., 2011). Although HML-2 expression has been linked with various pathological conditions, its transmembrane protein (TM) exerts immunosuppressive properties (Kämmerer et al., 2011). Therefore, its expression in villous and extravillous cytotrophoblast cells has been suggested to promote immune protection of the fetus during pregnancy (Kämmerer et al., 2011). HERVE has also been proposed as necessary component for placental development, as well as feto-maternal tolerance (Le Dantec et al., 2015; Kyriakou and Magiorkinis, 2023).

On the other hand, elevated expression of HERVs has been associated with autoimmune disorders, cancer, and neurological diseases. Among these diseases, multiple sclerosis (MS) stands out as the best-studied example. Kremer et al. (2019) have identified high levels of MS-associated retrovirus (MSRV) expression, a member of the HERVW family, in active brain lesions, cerebrospinal fluid (CSF), and peripheral blood of MS patients, with MSRV ENV mRNA and protein specifically detected in microglia and macrophages within these lesions. Other examples include rheumatoid arthritis, with *HERVK* RNA found in plasma collected from patients (Reynier et al., 2009) and amyotrophic lateral sclerosis (ALS), where elevated levels of both HERVK RNA and protein has been found in the brain of patients (Li et al., 2015). Beyond these observational findings, functional studies suggest a potential pathogenic role for MSRV ENV protein [now referred to as pathogenic envelope protein “pHERVW ENV” (Charvet et al., 2021)]. *In vitro* exposure of human microglia and macrophages to recombinant pHERVW ENV triggers a TLR4-dependent release of proinflammatory cytokines (Kremer et al., 2019). Similarly, in a primary rat coculture model, pHERVW ENV protein appears to activate microglia and promote their close physical interaction with myelinated axons, a process associated with axonal injury (Kremer et al., 2019). Moreover, the results of oligodendrocyte precursor cells treated with pHERVW ENV protein show impaired differentiation, pointing to a possible role of the protein in both inflammation and demyelination (Kremer et al., 2013). Functional studies in mice have provided compelling evidence that ENV proteins can actively contribute to disease pathogenesis. In a study investigating neuroinflammatory disease, the expression of pHERVW ENV protein in transgenic mice was shown to activate Toll-like receptor 4 (TLR4)-mediated innate immune pathways, leading to experimental allergic encephalomyelitis (EAE), a murine model of multiple sclerosis (Perron et al., 2013). Additionally, in a model of motor neuron disease, expression of HERVK ENV in the neurons of transgenic mice led to progressive motor dysfunction, neuronal loss in the spinal cord, and features resembling ALS (Li et al., 2015). The genomic origin of the original MS-derived MSRV RNA has been elusive, as its sequence is not present in genome assemblies, also complicating nomenclature. However, the MSRV-type envelope protein, pHERVW ENV, was recently shown to most probably originate from the ERVWE2 locus in chromosome X, and to be translated via ribosomal readthrough of mRNA with a stop codon (Brunel et al., 2024).

The results from recent studies have shown that altered expression of these retroviral elements is also associated with acute COVID-19 disease. RNA sequencing (RNA-Seq) analyses have revealed tissue-specific dysregulation of HERV loci in response to SARS-CoV-2, including upregulation in bronchoalveolar lavage fluid (BALF; Kitsou et al., 2021) and CD14+ monocytes from patients with COVID-19 (Koo and Morrow, 2024), as well as downregulation in peripheral blood mononuclear cells (PBMCs; Marston et al., 2021). Altered HERV expression in the nasal mucosa has also been associated with inflammatory mediator profiles and proposed as a potential biomarker for predicting COVID-19 severity (Petrone et al., 2023). In a subset of individuals with acute SARS-CoV-2 infection, the pHERVW ENV protein has been detected in peripheral blood T lymphocytes but is absent in T cells obtained from healthy donors (Balestrieri et al., 2021). Immunohistochemical analysis of postmortem tissues has identified pHERVW ENV protein in the lungs, heart, gastrointestinal tract, brain olfactory bulb, and nasal mucosa of patients with severe COVID-19 (Charvet et al., 2023). Moreover, both SARS-CoV-2 and spike protein exposure have been shown to induce the expression of *HERVW ENV* RNA and protein in cultured peripheral blood mononuclear cells (PBMCs) from a subset of healthy donors (Charvet et al., 2023).

Among the patients with COVID-19, pregnant women are one of the most vulnerable groups because of the changes in the immune system during pregnancy. In fact, COVID-19 disease has more severe manifestations in pregnant women (Villar et al., 2021), and has been associated with a higher risk for some pregnancy complications including hypertensive disorders of pregnancy (HDP) such as preeclampsia (PE) (Conde-Agudelo and Romero, 2022; Smith et al., 2023). PE is a fatal pregnancy disorder defined by maternal hypertension and proteinuria as a result of pregnancy (Rana et al., 2019). These pregnancy complications are associated with placental dysfunction, which may be directly induced or exacerbated by SARS-CoV-2 infection through increased placental inflammation, vascular damage, and impaired angiogenesis (Di Girolamo et al., 2021; Schwartz et al., 2023; Gabby et al., 2025).

Considering the upregulation of HERV levels in BALF and CD14 + monocytes from COVID-19 patients, this study aimed to investigate changes in the expression of retrotransposons in general and pHERVW ENV protein in particular, in placental villous tissue from women with COVID-19. We analyzed several (families of) retrotransposons associated with pulmonary COVID-19 disease (*HERVW*, *HERVH*; Kitsou et al., 2021) and others associated more generally with placental physiology (*ERVWE1*, *ERFVRD1, HERVE*, *HERVH*, *HEMO*; Mi et al., 2000; Noorali et al., 2009; Vargas et al., 2009; Heidmann et al., 2017). Both *HERVH* and *HERVK* were also included based on its previously reported expression in placenta (Kämmerer et al., 2011; Kong et al., 2024). We also selected genes whose altered expression is related to autoimmune and inflammatory disorders (*HERVW ENV, HERVK ENV*, and *HERVK POL*; Mameli et al., 2009; Li et al., 2015). We finally included *LINE-1* (Muñoz-Lopez et al., 2012), due to its high expression in the placenta and its involvement in early development and regulation of gene expression (Faulkner et al., 2009; Ma et al., 2025). Transcriptomic analysis also allowed us to analyze the ancient HERVL and HERVI families (Faulkner et al., 2009; Ma et al., 2025), as well as the lesser known HERV9 family, several of which are activated upon viral infection (Faulkner et al., 2009; Ma et al., 2025).

We studied the expression of these elements in placentas collected from pregnant women with COVID-19 according to the presence of SARS-CoV-2 in placenta or not, either by rigorously controlled qPCR, or using transcriptomics. Finally, we examined pHERVW ENV protein expression in placentas from both COVID-19 and non-COVID-19 pregnancies by immunofluorescence.

## 2 Materials and methods

### 2.1 Tissue samples and experimental design

In Zaragoza, placentas were collected from March 2020 to December 2021 (Facchetti et al., 2020; Medel-Martinez et al., 2023) in the Hospital Clínico Lozano Blesa (HCULB). Placental samples were collected according to the following inclusion criteria: SARS-CoV-2 infection during pregnancy (determined by a qPCR positive test from a nasal swab recorded in the medical history), age >18 years old and placenta tissue available for analysis (Fabre et al., 2021). In total, 32 placentas were analyzed, 14 out of those tested positive for SARS-CoV-2 assessed by RTqPCR (Fabre et al., 2021). Furthermore, additional preCOVID-19 placental samples were included. As non COVID-19 controls, 10 preCOVID-19 placental samples from uneventful pregnancies collected previously were included (Medel-Martinez et al., 2025). Furthermore, for some comparisons we also analyzed 10 preCOVID-19 placental samples from women diagnosed with PE provided by Hospital Clinic Barcelona, Spain.

We also analyzed the following placental samples collected at BSCH, Italy, from women with a SARS-CoV-2 positive test within 14 days before hospital admission (Facchetti et al., 2020): (a) three placentas exhibiting strong expression of SARS-CoV-2 nucleocapsid (NC) by immunoassays (Facchetti et al., 2020), referred to as SARS-CoV-2 positive; (b) three placentas without detectable expression of SARS-CoV-2 NC, referred to as SARS-CoV-2 negative. Another three placental samples collected before the COVID-19 pandemic were also included as controls. The demographic and clinical characteristics of the samples included in the qPCR analyses are shown in Supplementary Table S1, whereas the characteristics of the samples used for the pathological assessment are provided in Supplementary Table S2. To increase the sample size for transcriptomic analyses, a larger and partially distinct subset from the same experimental groups was used for RNA-seq, comprising 16 SARS-CoV-2–positive and 24 SARS-CoV-2–negative placentas (Supplementary Table S3).

### 2.2 Sample collection, tissue homogenization, RNA isolation, reverse transcription (RT) and quantitative polymerase chain reaction (qPCR)

Placental biopsies were obtained from the chorionic villous sites (placental parenchyma) carefully avoiding the placental bed. Placental sample collection, RNA preservation, tissue homogenization, RNA isolation including DNase treatment to remove potential genomic DNA contamination, reverse transcription and RT-qPCR was carried out following the protocols previously described (Oros et al., 2017; Schoorlemmer et al., 2020). PBMC isolation was carried out as described (Macías-Redondo et al., 2021).

cDNA was amplified using the primers and TaqMan qPCR assays detailed in Supplementary Table S4. Standard curves produced from serial dilutions were used to ascertain the efficiency of amplification (Supplementary Figure S1), minimal requirements for acceptance were R^2^ values of 1 ± 0.10 and efficiencies of 100 ± 10%. Only amplicons for which the melting curve showed a single peak were considered acceptable. No significant amplification was detected in controls. All reactions were carried out in triplicate and only measurements with a standard deviation <0.2 were considered.

Gene expression was recorded as Ct values. Fold change was calculated as 2^–∆∆Ct^ (Livak and Schmittgen, 2001) using *RPL13, RPL19* and *SDHA* as reference genes (Meller et al., 2005; Iglesias-Platas et al., 2014; Rydbirk et al., 2016). We also compared the expression between parallel RNA samples with or without reverse transcriptase (RTase).

### 2.3 Amplicon sequencing, library preparation, quantification and next generation sequencing

Products of selected qPCR reactions were purified with AmPure XP beads and analyzed with an TapeStation 4,200 Bioanalyzer (Agilent Technologies, USA). Libraries were prepared using the HyperPrep Plus Kit (KAPA Biosystems, USA), purified, quantified, and amplified using Illumina P7 and P5 primers.^1^ When necessary, specific fragments were further purified on a BluePippin platform (Sage Science, USA). Equimolar pools were sequenced on the MiSeq platform (Illumina, California) using a 100-cycle paired-end format.

Reference genomes were created using sequences from Supplementary Table S5 for every gene and trimmed fastq files were mapped to these reference sequences using local alignment with the Bowtie 2 program version 2.5.2. SAMtools version 1.20 was then used to extract the read counts and mapping statistics.

### 2.4 Transcriptomics

The corresponding fastq files were compiled, and we proceeded with their initial quality control (FastQC v0.11.9).^2^ The reads were further checked for adapter content and any needed trimming was performed with Trimmomatic (v0.39; Bolger et al., 2014). Filtered fastq reads, of minimum read length of 50 nucleotides, were then mapped against the Human Reference Genome (version GRCh38.p14) with the splice-aware aligner HISAT2 (v2.2.1; Kim et al., 2019). Read counts extraction per locus was achieved by the implementation of FeatureCounts (v2.0.6; Liao et al., 2014). More specifically, we measured the expression levels of five selected placenta-specific reference genes (RPN1, NRBP1, DHX15, DDX17, ARPC2), as indicated by the HRT Atlas v1.0 database (Hounkpe et al., 2021), and various HERV families (HERVK HML-1/HML-2/HML-3/HML-4/HML-5/HML-6, HERVW, HERVFRD, HERVH, HERVI, HERVL, HERVE, HERV9), whose integration coordinates in the human genome were extracted from the Telescope’s reference annotation (HERV_rmsk.hg38.v2; Bendall et al., 2019). The HML-2 solo LTR coordinates were identified based on the Subramanian et al. published annotation (Subramanian et al., 2011), which we converted from the hg19 to hg38p14 genome version with the UCSC Lift Genome Annotations Tool (Hinrichs et al., 2006). We also measured the read counts assigned specifically to syncytin-1 gene (*ERVWE1*). Normalization of each HERV family total read counts was performed using the average expression of the abovementioned five housekeeping genes (HKG) per sample. Statistical analysis of the differential expression results among the reported cases was performed in SPSS (v29.0). Log-transformed normalized counts per HERV family group were subjected to independent sample *t*-test (assuming unequal variance, two-tailed, with *p* < 0.05), to identify statistically significant expression differences between the selected groups tested.

### 2.5 Culturing and plasmid transfection in HEK293T cells

Plasmids used to express GFP, pHERVW ENV, or ERVWE1 have been described previously (Charvet et al., 2021) and the expressed proteins are encoded by cDNAs deposited in GenBank: AF331500.1 (pHERVW ENV) and AF208161.1 (ERVWE1).

HEK293T cells were cultured and transfected using Lipofectamine™ 2000 Transfection Reagent (Thermo Fisher Scientific, USA) according to standard procedures. To assess transfection efficiency, pMAX-GFP was co-transfected as a fluorescence control and detected in cells via fluorescence microscopy and flow cytometry (data not shown). After incubation (16- or 48-h post-transfection for pCMV-ERVWE1 and pCMV-HERVW ENV, respectively), cells were harvested, fixed, embedded in paraffin and sectioned as described for tissue sections.

### 2.6 Immunofluorescence by tyramide signal amplification

HEK293T cells were fixed in 4% neutral buffered formaldehyde, dehydrated through a graded ethanol series, cleared with xylene, and subsequently infiltrated and embedded in paraffin. Placental collection, fixation in 10% buffered formalin, weighing, trimming of the cord and membranes, sampling and examination were performed according to the Amsterdam guidelines, as described (Facchetti et al., 2020).

Section (two-micron thick) were deparaffinized and rehydrated in graded solutions of ethanol and distilled water. Endogenous peroxidase was blocked during rehydration and antigen retrieval was performed after it.

We used the monoclonal antibodies GN_mAb_Env03 and GN_mAb_Env16 [provided by GeNeuro; (Charvet et al., 2021)] to detect the HERVW ENV and ERVWE1 proteins, respectively. Immunostaining was performed using GN_mAb_Env03 (1:12.000. GeNeuro, Switzerland), GN_mAb_Env16 (1:4.000. GeNeuro, Switzerland), HCGβ (clone EPHCGR2, 1:100. Abcam, UK, #ab131170), and anti-SARS-CoV-2 Nucleocapsid (polyclonal rabbit, 1:4.000. Sino Biological, China, #40588-T62).

Polymer amplifications were followed by tyramide fluorophore addition AF488 (Thermofisher Scientific, USA #B40953) CF555 (Biotium, California #96021) and CF647 (Biotium, California #96022). After the first immune reaction, the second and third reactions were visualized by combining different tyramides. VECTASHIELD Antifade Mounting Medium with DAPI (vector, #H-1200) was used to contrast and mount immunofluorescence slides.

### 2.7 Imaging acquisition: microscopic and digital analysis

Placental sections were digitized using Axioscan7 (Zeiss) at 20x magnification using Colibri 7 lamp as LED light source with different illumination wavelength (450–488; 540–570; 615–648) depending on different module of the LED (385 nm, 475 nm, 567 nm and 630 nm). Digitized slides were analyzed using QuPath-0.4.3, DAPI signals were used as nuclear markers to detect the cells and then a single measurement classifier was trained to detect NC, HERVW ENV and HCGβ positivity. At last, a composite classifier was used to identify the different cell populations.

### 2.8 Statistical analysis

All statistical analyses and graphical representations were performed using Prism 9 (GraphPad Software, California, USA), with a significance level of *p* < 0.05. Normality was assessed using the Shapiro–Wilk test (*n* < 50). For continuous variables, normally distributed data were analyzed using the Student’s *t*-test for two-group comparisons or one-way analysis of variance (ANOVA) for more than two groups, applying Welch’s correction when variances were unequal; non-normal data were analyzed using the Mann–Whitney U test for two-group comparisons or the Kruskal–Wallis test for multiple-group comparisons. Categorical variables were compared using the Chi-square test, or Fisher’s exact test when any group contained fewer than five cases. XY correlation analyses were performed using Pearson or Spearman tests depending on data distribution. The Multiple of the Medians (MoM) method was used to assess the distribution and variability of gene expression levels across experimental conditions.

## 3 Results

### 3.1 Quality control of qPCR amplicons for PCR-based expression analysis

COVID-19 disease caused by SARS-CoV-2 disease has been associated with altered expression of retrotransposons and HERVs (Marston et al., 2021; Charvet et al., 2023; Grandi et al., 2023). Expression of such elements is reportedly high in placenta, and contributes to maintenance and development of this tissue (Muir et al., 2004). To evaluate the possibility that SARS-CoV-2 infection modulates placental expression of TEs (transposable elements), HERVs and of *HERVW ENV* in particular, we compared expression in samples taken from preCOVID-19 uneventful pregnancies (healthy controls) and mothers who tested positive for SARS-CoV-2 during pregnancy. As placental infection with SARS-CoV-2 has been described (Fabre et al., 2021; Fahmi et al., 2021; Radan et al., 2024), we also compared HERV expression in SARS-CoV-2 positive and negative placenta. As pHERVW ENV protein and RNA had been shown to be directly induced by SARS-CoV-2 in a subset of PBMC cultures (Charvet et al., 2023), we paid special attention to different *ENV* variants expressed.

Within the HERVW multicopy family, the *ERVWE1* gene stands out as it has been co-opted to carry out specific functions in the placenta, especially in syncytiotrophoblast (STB) formation. We employed an assay termed ERVWE1 to specifically detect transcripts from this gene. We will refer to RNAs produced from the combined *HERVW* loci (not including *ERVWE1)* as *HERVW ENV,* as suggested previously (Charvet et al., 2021). To determine expression of these sequences, we employed two complementary assays (see Supplementary Table S4). Both assays, termed HERVW ENV [also referred as UNO in (Levet et al., 2017) and MSRV ENV (Mameli et al., 2009)], specifically detect *HERVW ENV*, in the latter assay based on a unique 4 amino-acid deletion in *ERVWE1* not present in any other copy of HERVW including *MSRV* (Mameli et al., 2009).

We started with a detailed analysis of the amplification properties of all assays, including assessment of minimal amplification efficiency, the amplification of single products etc. as detailed in M&M. To make sure that amplification was dependent on reverse transcription, and did not originate from contaminating genomic endogenous retroviral DNA sequences, amplification was evaluated following exposure of RNA samples to the RT step with or without RTase. As shown in Supplementary Figure S2, all HERVs exhibited at least a 100-fold difference in amplification signal between the two conditions (+/− RTase), ensuring that HERV measured expression was primarily derived from cDNA.

To assess selectivity of the amplicons, amplified products of all assays were sequenced, resulting reads were aligned to a set of reference sequences (Supplementary Table S5). Results showed that > 96% of reads mapped to the intended TEs (Supplementary Table S6). Since several of our assays (ERVWE1, HERVW ENV, and MSRV ENV) targeted closely related sequences derived from the HERVW family, we further evaluated cross-mapping, assessing the proportion of reads that mapped to non-target sequences. A 100% of the reads generated from the products amplified by the HERVW ENV assay (Levet et al., 2017) aligned to both the *HERVW* provirus and the MSRV ENV sequence, with minimal cross-mapping to *ERVWE1* (0.23%). The same applies to the MSRV ENV assay (Mameli et al., 2009), except for a 1.4% of reads cross-mapping to *ERVWE1* (Supplementary Table S7).

### 3.2 HERVs in placentas delivered by pregnant women with COVID-19

To establish the basal expression levels of retro-transposable elements in placenta, expression of each element was also compared simultaneously in placental samples and PBMCs derived from healthy donors, using the validated assays (Supplementary Figure S3). The comparison performed between PBMCs and placenta confirmed the high expression levels of HERVs in placenta (Chuong, 2018) as compared to PBMCs (*ERVWE1*: 391.22, *p* < 0.0001. *ERVFRD1*: 27.19 fold, *p* < 0.0001. *HERVW*: 172 fold, *p* = 0.0006; *MSRV*: 5.66 fold *p* < 0.0001; *HERVK ENV*: 3.83 fold, *p* = 0.0006; HERVK *POL*: 3.32 fold, *p* = 0.0012; *LINE-1*: 4.75 fold, *p* = 0.0012; *HEMO*: 1130 fold, *p* = 0.0029; *HERVH POL*: 17.27 fold, *p* = 0.0006) and helped us to establish placental basal expression in healthy placentas.

**Figure 1:**
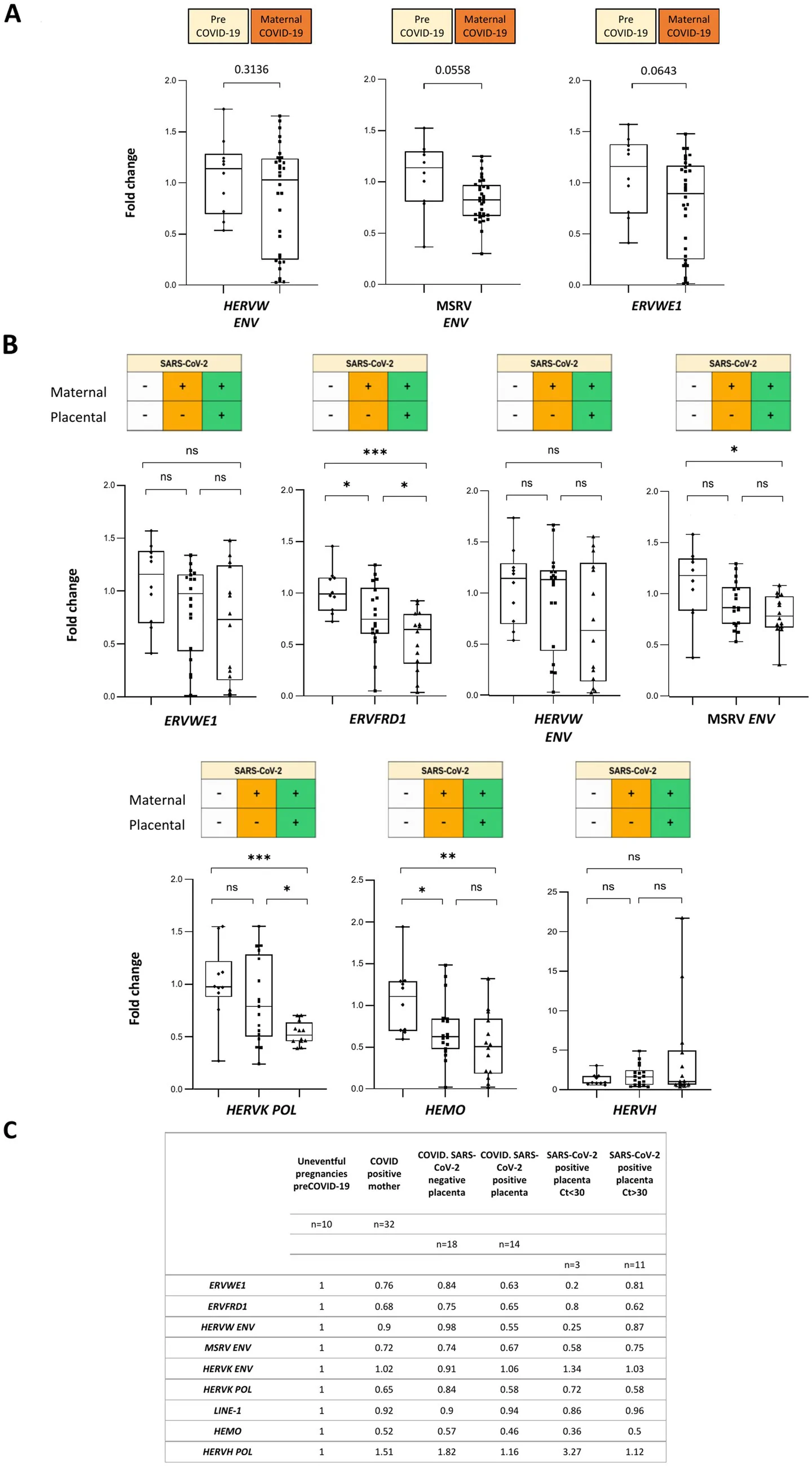
Analysis of retrotransposon expression in placental samples from women with COVID-19 during pregnancy using qPCR. Women who were qPCR-positive for SARS-CoV-2 (*n* = 32) were separated into two groups, either with a positive test in placenta (*n* = 14) or not (*n* = 18). The latter group was further divided according to the *Ct* value obtained (*Ct* < 30; *n* = 3; *Ct* > 30; *n* = 11). The presence of SARS-CoV-2 in placenta was measured by standard diagnostic qPCR (see M&M). Expression was compared to a group of preCOVID-19 placentas (*n* = 10) delivered by women with uneventful pregnancies. Box plots showing median expression and interquartile ranges (IQR) of placentas from **(A)** all SARS-CoV-2-infected pregnant women (*n* = 32) compared with preCOVID-19 uneventful controls (*n* = 10) and **(B)** women who were qPCR-positive for SARS-CoV-2 (*n* = 32) separated into two groups, either with a positive test in placenta (*n* = 14) or not (*n* = 18). The statistical significance of the differences defined by the *p*-value is indicated: *p* < 0.05 (*); *p* < 0.01 (**); *p* < 0.001 (***); *p* < 0.0001 (****). **(C)** The table shows the data recalculated as fold expression compared to the median of expression in the preCOVID-19 placentas. Fold change of controls (preCOVID-19 placentas) is set to 1.

We next examined TE expression in placental samples from pregnant women with gestational COVID-19, compared to preCOVID-19 samples from uneventful pregnancies. *ERVWE1*, *HERVW ENV*, and *MSRV ENV* expression levels were lower in case of maternal COVID-19 (Figure 1A) reaching borderline statistical significance for *ERVWE1* (*p* = 0.064) and MSRV *ENV* (*p* = 0.0558). We next continued the analysis comparing preCOVID-19 samples taken from uneventful pregnancies (*n* = 10) with either SARS-CoV-2 positive or negative placentas (Figure 1B). With the exception of HERVH (see below), lower levels of expression were observed when analyzing several other retroviral elements genes and elements, including *ERVFRD1*, *HERVK ENV*, *HERVK POL*, *LINE-1*, *HEMO* (Figures 1B,C and Supplementary Table S8).

*ERVWE1* expression levels were lower in SARS positive placentas (0.63 fold) and negative placentas (0.81 fold), compared to preCOVID-19 SARS-CoV-2 negative pregnancies (control group; Figures 1B,C). While these changes were not significant, they indicated a trend toward lower expression comparing positive placentas to the control group (*p* = 0.060). *HERVW ENV* relative expression levels determined by HERVW ENV assay (Levet et al., 2017) were lowered in SARS-CoV-2-positive placentas (0.55 fold) compared to healthy controls; however, this difference did not reach statistical significance (*p* = 0.2125), despite a notable trend. No significant alterations were observed in SARS-CoV-2-negative placentas relative to healthy controls. Placental *HERVW ENV* expression levels determined by the MSRV ENV assay (Mameli et al., 2009) were 0.67 and 0.74 fold lower in positive placentas and negative placentas (*p* = 0.0118 and 0.0716, respectively), when compared to the control group. At the same time, *ERVFRD1* levels of positive and negative placentas presented a lower relative expression (0.65 fold and 0.75 fold respectively) reaching significance in both cases (control VS positive placentas *p* = 0.0003; control VS negative placentas, *p* = 0.0406). The relative expression of *HERVK POL* was also lower in positive placentas (0.58 fold, *p* = 0.0003). Likewise, relative expression levels of *HEMO* were significantly lower in positive (0.46 fold, *p* = 0.0029) and negative placentas (0.57 fold, *p* = 0.029) compared to preCOVID-19 controls. We also analyzed if there was any correlation between placental SARS-CoV-2 load and HERV expression. We took into account the level of SARS-CoV-2 expression in positive placentas, dividing them into two groups: high levels (Ct < 30) and low level (Ct > 30; Figure 1C and Supplementary Table S8). In the high expression group, *HERVW ENV*, *ERVWE1 and HEMO* levels were extremely low (0.25 fold, *p* = 0.0070 and 0.20 fold, *p* = 0.0280, 0.36 fold, *p* = 0.0069 respectively). These results show that expression of *ERVFRD1, HEMO, ERVWE1*, *HERVW ENV* and *MSRV ENV* is negatively affected by maternal COVID-19 disease and especially by the presence of placental SARS-CoV-2. In the case of HERVH, our data showed significantly higher levels when analyzing placentas with high viral load (3.27 fold, *p* = 0.5734). Moreover, we were unable to establish a significant correlation between SARS-CoV-2 RNA levels and HERV expression levels, when performing correlation tests (Supplementary Figure S4).

**Figure 2:**
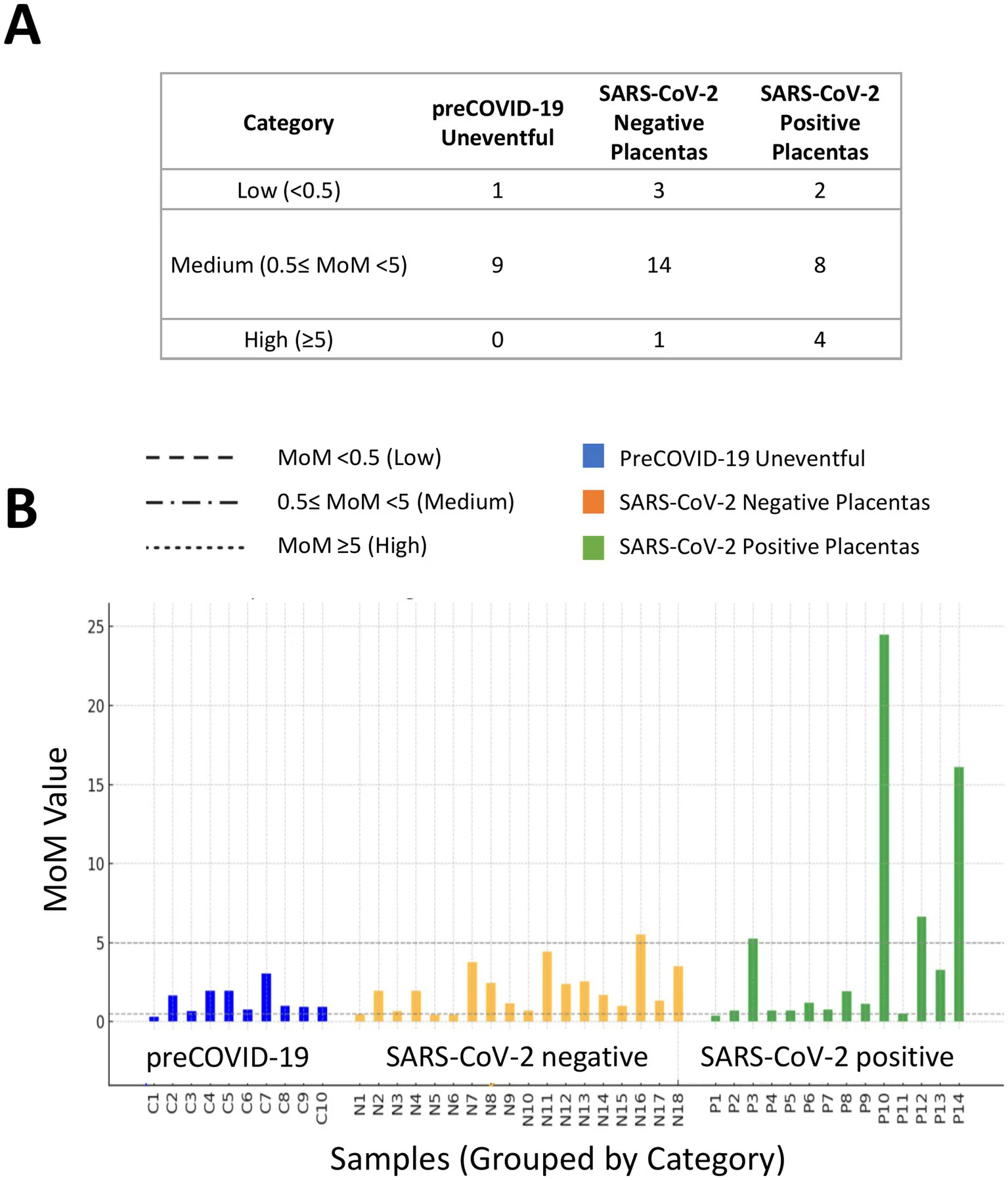
Analysis of *HERVH* expression by multiples of the median (MoM) test. Comparison of pre-COVID-19 uneventful pregnancies (blue), and women with COVID-19 disease during pregnancy (divided according to SARS-CoV-2 presence in placenta, yellow/ochre and green for negative and positive, respectively). **(A)** MoM values were calculated by dividing the fold changes for each sample (Figure 1) by the median fold change of the pre-COVID-19 group. Each bar corresponds to the MoM value of an individual sample. **(B)** Samples were categorized according to the following criteria: MoM < 0.5 as this represents repression associated with COVID-19 disease; 0.5 < MoM < 5; MoM > 5 as an arbitrary indicator of highly induced expression. The number of samples in each category was counted for each group. The difference between the three groups showed no statistical significance (*χ*^2^ test).

*HERVH* levels showed a trend toward overexpression in placentas delivered by women with COVID-19 (1.51 fold) compared to the control group. However, we could not find an association between the presence of SARS-CoV-2 in placenta and *HERVH* upregulation by pairwise comparisons (control VS negative placenta 1.82 fold, *p* = 0.465; control VS positive placenta 1.16 fold, *p* = 0.8859). As *HERVH* expression found was highly variable and upregulation was observed in only a few samples we studied the deviation of test results individually. We found 5 samples with higher *HERVH* expression using >5 MoM as a cut-off, from which 4 were SARS-CoV-2 positive in placental tissue (Figure 2), suggesting that HERVH expression was strongly induced in a subset of SARS-CoV-2 positive placentas.

### 3.3 Transcriptomic analysis of HERV expression in placentas

**Figure 3:**
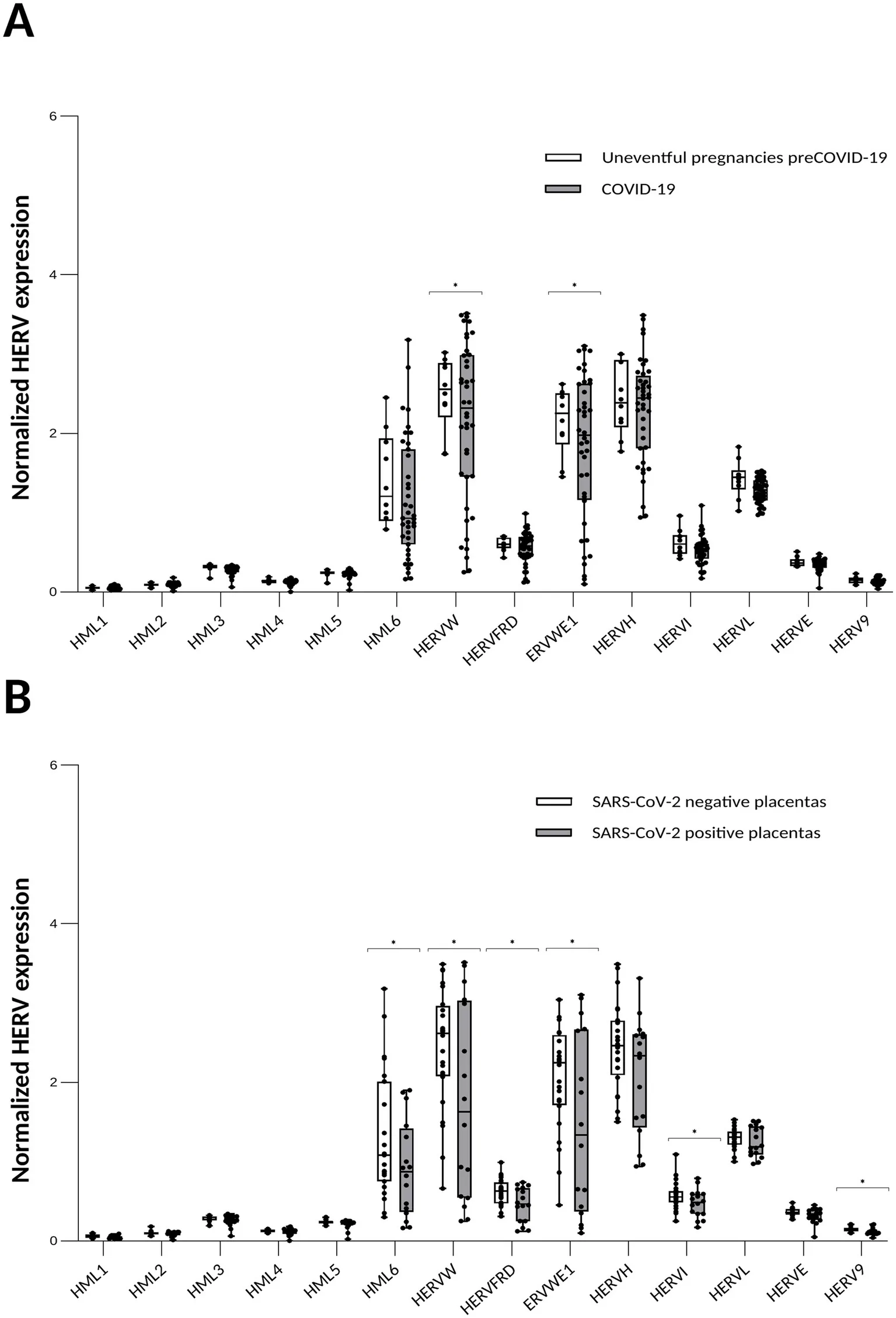
Transcription levels of human endogenous retroviral (HERV) families in placental samples. Placenta RNA-Seq reads were mapped to the human reference genome (GRCh38p14) and HERV loci were identified to quantify aligned reads at the locus level. Reads were aggregated at the family level and normalized using placenta-specific reference genes (*RPN1*, *NRBP1*, *DHX15*, *DDX17*, and *ARPC2*). Expression values were recalculated and represented at the HERV family level, except for *HML1-6* and *ERVWE1*. **(A)** Comparison of placental samples collected from women infected by SARS-CoV-2 during pregnancy (*n* = 40) with samples collected from uneventful pre-COVID-19 pregnancies (*n* = 10). **(B)** Comparison of placental samples from pregnancies affected by COVID-19 disease with detectable virus in the placenta (*n* = 16) vs those with undetectable SARS-CoV-2 genome in placental tissue (*n* = 24).

A slightly different set of RNA samples from the same experimental groups (Supplementary Table S9), was subjected to sequencing. The uneventful preCOVID-19 control samples had been sequenced previously (Medel-Martinez et al., 2025). We mined the resulting RNA-Seq datasets to analyze differential expression of TEs at the family level (separating HERVK elements into HML1-6), except for *ERWE1* and *ERVFRD1 loci*, which were analysed individually. Results regarding differences between different groups and their statistical significance are detailed in Supplementary Table S10. While the analysis revealed few significant changes in HERVs transcription levels between expression in samples taken from preCOVID-19 uneventful pregnancies (healthy controls) or mothers who tested positive for SARS-CoV-2 during pregnancy, the trends toward somewhat lower levels of several HERV families was evident (Figure 3). This holds for *HERVFRD* (0.92 fold, *p* = 0.073), *HERVI* (0.85 fold, *p* = 0.080) and *HERVL* (0.89 fold, *p* = 0.080). This same analysis did not reveal changes in *HERVE*, *HERV9* and *HERVK* levels (Supplementary Table S10). Similar to the RTqPCR analysis, the transcriptomic analysis showed placental repression of HERVW upon COVID-19 infection. This was evident for both the *ERVWE1* locus individually (0.85 fold, *p* = 0.014), and the combined HERVW *loci* (0.86 fold, *p* = 0.019; Figure 3A and Supplementary Table S10).

We also mined the RNAseq datasets to compare placenta samples that expressed the SARS-CoV-2 genome (SARS-CoV-2 positive), to placentas negative for the presence of viral RNA (preCOVID-19 control samples and negative placentas collected in 2020 (*n* = 24) combined). Interestingly, SARS-CoV-2 positivity was significantly correlated with downregulation of some HERV groups, namely HERVK (HML-6), HERVW, HERVFRD, HERVI and HERV9 as well as the *ERVWE1* locus itself (Figure 3B). These results mostly confirm data obtained by qPCR, and add *HERVK* (*HML-6*), *HERVI* and *HERV9* to the group of HERVs downregulated as a result of COVID-19 disease.

### 3.4 Expression of HERVs in placentas delivered by pregnant women suffering from HDP

We had previously reported that the presence of placental SARS-CoV-2 in women susceptible to hypertensive disorders of pregnancy (HDP) may contribute to the onset of the disorder. As a matter of fact, a high percentage of placentas that tested positive for SARS-CoV-2 were delivered by mothers suffering from HDP (Supplementary Table S1 and Fabre et al., 2021). Among the 14 SARS-CoV-2 positive placentas previously analyzed, 9 belonged to the HDP group and 5 to the no HDP group.

We then analyzed SARS-CoV-2–positive placentas according to HDP status. In SARS-CoV-2–positive placentas from women without HDP, transcript levels of *ERVWE1. ERVFRD1* (0.06 fold, *p* = 0.0047; 0.25 fold, *p* < 0.0001 respectively), *HERVW* ENV and *HEMO* (0.06 fold, *p* = 0.0080; 0.11 fold, *p* = 0.0006 respectively) were significantly much lowered in the positive placentas that were not delivered by women suffering from HDP when compared to preCOVID-19 placentas collected from uneventful pregnancies. *HERVH* levels were also significantly higher in positive placentas of women without HDP when compared with preCOVID-19 healthy placental samples (5.24 fold, *p* = 0.1469). Results are shown in Supplementary Table S11.

Given that altered expression of HERV-derived placental genes, including syncytins, has previously been associated with preeclampsia (Vargas et al., 2011; Wang et al., 2024), preeclampsia (PE) placental samples collected during the preCOVID-19 era were also included in this HDP-focused analysis as a separate disease-control group. Using preCOVID-19 placentas from uneventful pregnancies as the reference group, preCOVID-19 PE placental samples showed a similar trend toward lower expression of several HERV-derived placental genes, including *ERVWE1* (0.59 fold, *p* = 0.0376), *ERVFRD1* (0.47 fold, *p* = 0.0028), *HERVW ENV* (0.63 fold, *p* = 0.0640), *HERVK POL* (0.48 fold, *p* = 0.0028), and *HEMO* (0.40 fold, *p* = 0.0091), while *HERVH POL* showed a higher fold-change value (1.44 fold, *p* = 0.8501; Supplementary Table S11).

Overall, placental samples collected from women with preCOVID-19 PE showed, as expected, a comparable trend toward lower expression of several HERV-derived placental genes relative to placentas from uneventful pregnancies. However, the marked downregulation observed in SARS-CoV-2–positive placentas from women without HDP suggests that the presence of SARS-CoV-2 in the placenta is associated with a PE-like HERV expression signature, irrespective of the subsequent clinical development of HDP.

### 3.5 HERVW ENV protein in COVID-19 placentas

Both RT-qPCR and transcriptomic analysis showed generally lower levels of HERV expression in placentas delivered by women who suffered an episode of COVID-19 disease during pregnancy (Figures 1, 3). As pHERVW ENV is induced in several tissues as a response to SARS-CoV-2 infection (Balestrieri et al., 2021; Charvet et al., 2023) we examined the presence of pHERVW ENV protein in placentas by immunofluorescence.

**Figure 4:**
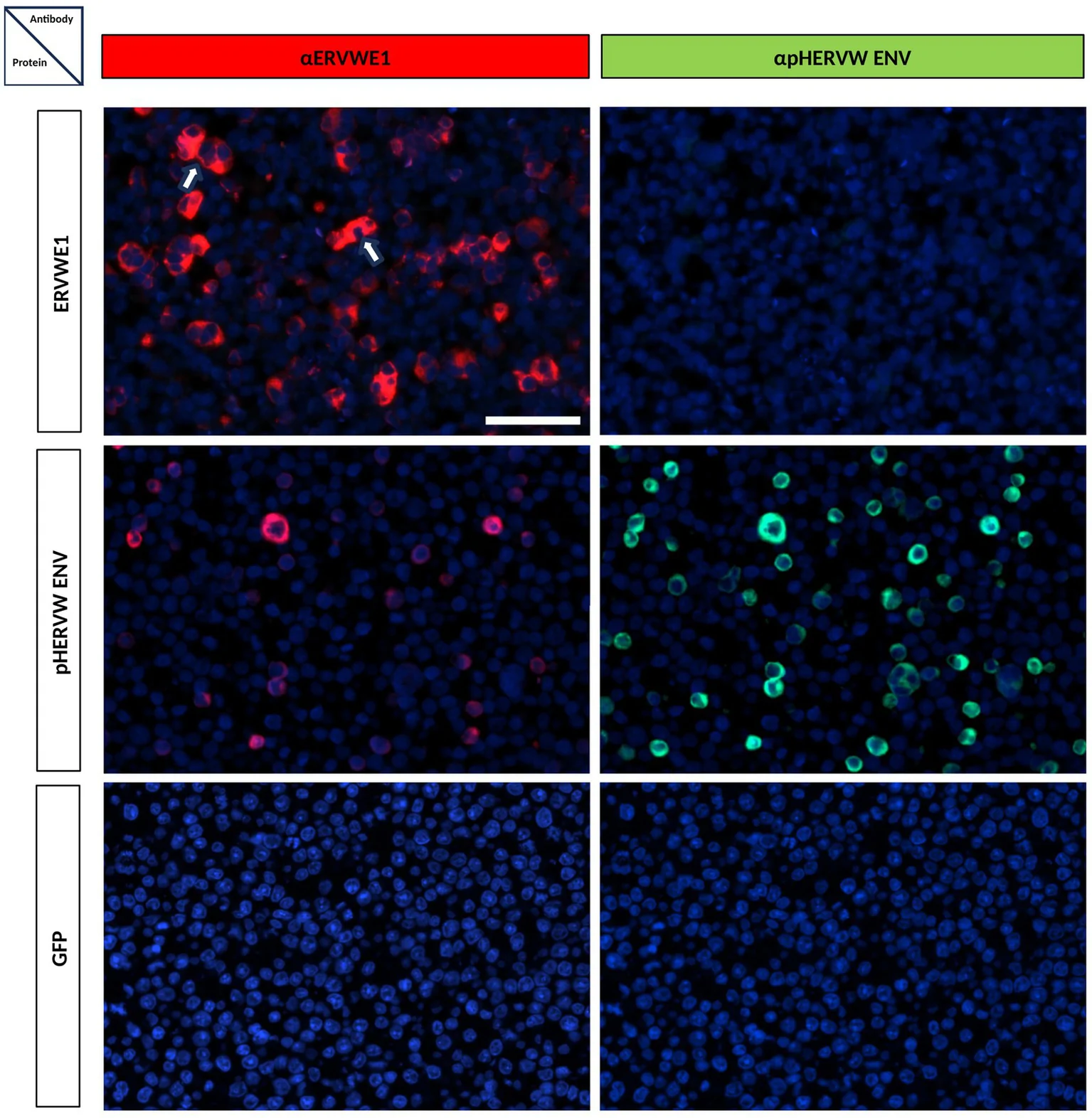
Characterization of antibody specificity. Plasmids directing expression of pHERVW ENV (pathogenic envelope protein) or ERVWE1 proteins were transfected into HEK293T cells. Slides were co-stained with ɑERVWE1 and ɑpHERVW ENV antibodies, overexpressed proteins were detected by multiplex tyramide signal amplification immunofluorescence (TSA-IF). Cell nuclei were stained with DAPI (blue). Images were acquired in a single-channel mode using magnification 100X. Cells transfected only with a GFP-expressing plasmid were used as a negative control. Scale bar: 80 μm. Note that forced expression causes syncytium formation (see arrows).

Since pHERVW ENV and ERVWE1 proteins differ by only a few amino acids and antibody cross-reactivity was anticipated, we first established experimental conditions that enabled specific detection of pHERVW ENV in transfected cells. To this end, double immunofluorescence with tyramide signal amplification (TSA-IF) was employed to simultaneously detect both antigens. Staining was optimized for cells transfected with plasmids driving expression of either pHERVW ENV or ERVWE1. The *α*ERVWE1 antibody easily detected transfected ERVWE1, as opposed to mock-transfected cells (Figure 4, left panels). Unfortunately, signal was also detected in some cells transfected with pHERVW ENV. Conversely, the αpHERVW ENV antibody (GN_mAb_Env03), easily and specifically detected transfected pHERVW ENV, producing no detectable signal in ERVWE1-expressing cells (Figure 4, right panels).

**Figure 5:**
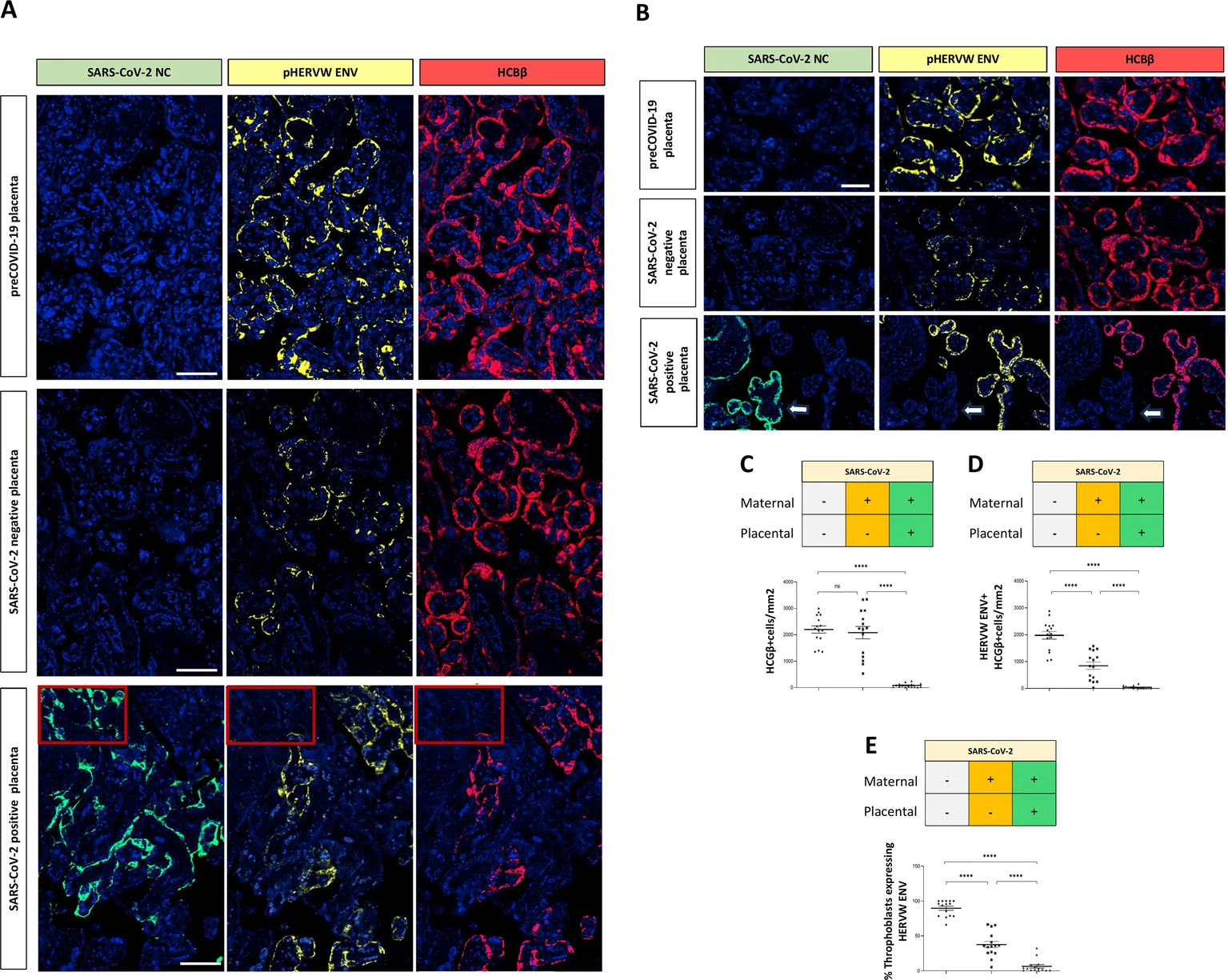
Reduced presence of pHERVW ENV protein after SARS-CoV-2 infection. Placental sections were co-stained with antibodies targeting SARS-CoV-2 nucleocapsid protein (NC), HCGb, and pHERVW ENV protein. Staining was detected by multiplex tyramide signal amplification immunofluorescence (TSA-IF). Nuclei were stained with DAPI (blue). **(A)** Representative microscopy images are shown of stains carried out on sections from preCOVID-19 placentas (*n* = 3), placentas collected after pregnancies affected by COVID-19 disease, with either a SARS-CoV-2 positive test in placenta (*n* = 3) or not (*n* = 3). NC-staining was not homogeneous throughout sections, images with NC-positive cells have been selected (see squares). Note that even in selected areas, NC-positive and negative cells coexist. Images are acquired in single-channel mode using magnification 60×. Scale bar: 100μm. **(B)** Representative areas of the synctiotrophoblast cells positive for pHERVW ENV in each group. **(C)** Quantification of HCGb positive cells per mm^2^. **(D)** Quantification of synctiotrophoblast cells positive for both HCGb and pHERVW ENV per mm^2^. **(E)** Quantification of pHERVW ENV-expressing synctiotrophoblasts. The ratio between cells positive for pHERVW ENV and the number of synciotrophoblasts is expressed as a percentage. Five random fields per placental section were automatically selected and synctiotrophoblast cells were counted in single channel mode.

Using the conditions established, we subsequently tried to detect pHERVW ENV protein in placental tissue and compared placentas from preCOVID-19 pregnancies, with placentas derived from COVID-19 pregnancies. Since SARS-CoV-2 has been reported to infect the STB (Facchetti et al., 2020; Hosier et al., 2020; Garrido-Pontnou et al., 2021), we also used human chorionic gonadotropin beta (HCGβ) as a marker to identify this cell layer in placentas (Beck et al., 1986; Cole, 2010; Aldaz-Carroll et al., 2015). Immunostaining for pHERVW ENV protein showed positivity across all analyzed samples, with pHERVW ENV signal detectable exclusively in the STB, as determined by cellular morphology and co-staining with hCGβ (Figure 5). Visual inspection indicated that in preCOVID-19 placentas most if not all hCGβ+ syncytial trophoblast cells stained also positive for pHERVW ENV. In the placentas collected from SARS-CoV-2-positive mothers without placental SARS-CoV-2 infection (confirmed by the absence of NC signal), we observed syncytial trophoblast cells that similarly co-stained for hCGβ and pHERVW ENV proteins (Figure 5A). The number of hCGβ-positive syncytial cells was not affected by maternal COVID-19 disease (Figures 5B,C). However, the number of ENV+/ hCGβ+ cells (counted per mm2) was significantly lower compared to the nonCOVID-19 controls (Figures 5B,D). In addition, the total number of ENV + trophoblast cells was significantly reduced compared to preCOVID-19 controls (Figure 5E). Based on this combined data, we conclude that maternal COVID-19 disease downregulates placental pHERVW ENV protein levels, in the absence of STB damage.

We analyzed placentas that tested positive for SARS-CoV-2, as determined by immunofluorescence using α-nucleocapsid antibodies, which detected NC protein in the cytoplasm of STB cells (Facchetti et al., 2020). In SARS-CoV-2 NC-positive placentas, we observed NC-positive cells as DAPI-positive cells with syncytial morphology which were however devoid of hCGβ signal (see square in Figure 5A and the arrow in Figure 5B). In addition to the absence of hCGβ signal, no ENV staining was visible either (Figure 5B). Quantification of staining in randomly selected fields showed a near absence of ENV+/ hCGβ+ cells upon placental SARS-CoV-2 infection. This was true when cells were quantified per mm2 (Figure 5D) or based on syncytial morphology of the cells (Figure 5E). This was mainly due to the almost total absence of hCGβ+ syncytial cells (Figure 5C).

Together, these data indicate that maternal COVID-19 disease is associated with reduced staining of placental pHERVW ENV protein, even in the absence of evident STB damage. This interpretation relies on antibody specificity validated in transfected cells; however, such validation does not inherently confirm that this specificity is maintained in tissue sections. Nevertheless, given the lack of cross-reactivity observed even under conditions of high syncytin-1 (ERVWE1) overexpression, we favor the interpretation that our results reflect the specific downregulation of pHERVW ENV.

## 4 Discussion

### 4.1 COVID-associated downregulation of HERV in placental parenchyma

We studied expression of retrotransposons in placentas delivered by mothers who had suffered an episode of COVID-19 disease during pregnancy. Expression of multiple HERVs was analyzed by mining data from RNAseq datasets and assayed by qPCR assays, which were rigorously tested for efficiency, and specificity. The latter included inspection of melting curves and sequencing of PCR products. As HERVW was found to be downregulated in the placental villous tissue, we assessed the presence of pHERVW ENV protein in placentas by immunofluorescence. Using both approaches, we evaluated the impact of maternal COVID-19 disease by comparing cases both with and without evidence of placental SARS-CoV-2 expression.

Combining results from optimized and fully characterized qPCR assays and transcriptomic analysis, our analysis revealed an overall trend toward reduced placental HERV expression in mothers with COVID-19. Downregulation of several elements was specific (*ERVFRD1, HEMO*, *HERVK*), as many other HERVs (such as *LINE-1*, *HERVL* and *HERVE*) elements were not significantly altered as a result of maternal COVID-19 disease (Figures 1, 3). Looking at the combination of qPCR or transcriptomics, HERVW expression trends lower in cases of maternal COVID-19 disease, reaching statistical significance for *HERVW* and *ERVWE1* (Figure 3A). Overall downregulation of HERVs was exacerbated by placental SARS-CoV-2, including lower expression of HERVW elements.

Downregulation also affected *HERVH* expression in most of our samples. However, in five cases, four of which tested positive for placental SARS-CoV-2, *HERVH* was upregulated. Such a selective induction limited to responsive samples, is reminiscent of SARS-CoV-2 induced induction of HERVW in PBMC from a subset of healthy donors only (Charvet et al., 2023).

In contrast to SARS-CoV-2 infection-induced activation of Human Endogenous Retroviruses (HERVs) in multiple cell types (Balestrieri et al., 2021; Kitsou et al., 2021; Charvet et al., 2023; Petrone et al., 2023), we found no such activation in placental villous tissue. Our study revealed a general downregulation of HERVs in placental tissues from pregnant women with COVID-19. Our observations are in accordance with recent data suggesting that SARS-CoV-2 promotes dysregulation and even downregulation of many retrotransposons in at the MFI (maternal-fetal interface), along with changes in the chromatin landscape (Gao et al., 2023).

### 4.2 Cohort timing and placental SARS-CoV-2 infection

In contrast to our results, a recently published competing study (Yoshida and Ohtani, 2025) reported a general upregulation of LTR retrotransposons and endogenous retroviruses, although no upregulated HERVW was mentioned. The discrepancy between our results may stem from cohort timing. While they exclusively analysed placentas from mothers who tested positive for SARS-CoV-2 at delivery, half of our samples involved infections during early-mid-pregnancy. Consequently, we interpret that acute infection may trigger generic HERV upregulation, whereas our samples from earlier-stage infections exhibit generic downregulation. According to our data during or after recovery from acute infection, over time placental HERV levels become downregulated. A distinct mechanism operates in case of persistent infection of the placenta. The consistently low expression in SARS-CoV-2-positive placentas indicates that the observed downregulation is driven by the persistent infection itself, independent of gestational timing. The most significant finding, is, that we did observe HERVW downregulation at term, suggesting that maternal SARS-CoV-2 exposure may induce long-term alterations in placental gene expression, potentially affecting placental development and function during critical early windows of fetal development.

### 4.3 HERVW pENV protein in COVID-19 placentas

We tested the cross-reactivity of αpHERVW ENV and αERVWE1 antibodies in transfected cells to establish conditions under which pHERVW ENV detection was selective. No cross-reactivity with ERVWE1 was observed, even when highly over-expressed. We therefore proceed on the premise that our anti-pENV antibody does not cross-react with ERVWE1. This assertion is qualified by the assumption that this specificity is preserved in tissue sections. As single-antigen conditions in healthy tissue do not exist, exclusive recognition of pHERVW ENV cannot be formally demonstrated in human tissue sections. We detected pHERVW ENV protein in the syncytiotrophoblast (STB) of both preCOVID-19 and COVID-19 placentas, though expression was reduced following maternal COVID-19 disease, even in SARS-CoV-2 negative placentas. Almost complete loss of protein correlated with syncytial damage linked to placental SARS-CoV-2 (see Figure 5).

The pHERVW ENV protein has been classified as pathogenic (Levet et al., 2017; Charvet et al., 2021) due to its ability to activate innate immune responses and induce pro-inflammatory cytokines (Kremer et al., 2019; Ruberto et al., 2025). As in this study we confirm that both *HERVW ENV* transcripts and pHERVW ENV protein are expressed at high levels in placenta, inflammatory immune responses must be either attenuated in placenta, or TLR4 activation and downstream signaling pathways may be physiologically relevant in placenta (Koga and Mor, 2010; Firmal et al., 2020). These placenta-specific mechanisms may implicate specific HERV-W copies. This possibility aligns with our group’s previous work demonstrating that distinct HERV copies are elevated in ALS (Moreno-Martinez et al., 2024) and MS (Macías-Redondo et al., 2021) in the absence of generalized overexpression. Consequently, it may be that the identity of the expressed copies, rather than their total expression levels, that dictates (patho) physiological outcomes.

### 4.4 Mechanistic interpretations

Significant histopathological changes such as villitis, syncytial knotting, trophoblast necrosis, and increased perivillous fibrin deposition associated with damage to the STB, have been detected in placentas from pregnant women with COVID-19 (Garrido-Pontnou et al., 2021; Gabby et al., 2025). Our combined data are consistent with another syncytial alteration in placentas from SARS-CoV-2-positive women, notably reduced ENV expression, even in the absence of demonstrable placental SARS-CoV-2 (Figure 5, panel B). While maternal COVID-19 did not alter hCGβ localization or the count of hCGβ-positive syncytial cells, these metrics offer an incomplete view of STB integrity. Consequently, we cannot rule out that subclinical alterations, including ENV depletion or other unexamined factors, could have compromised the syncytial barrier and facilitated SARS-CoV-2 infection. The presence of syncytial SARS-CoV-2 is associated with widespread STB disruption, evidenced by the simultaneous loss of hCG*β* and HERVW ENV signals in areas positive for NC (Figures 5A,B). While downregulated HERV expression may suggest a role for HERV dysregulation in placental dysfunction, our data cannot determine whether reduced HERV expression reflects a direct effect of SARS-CoV-2 on HERV transcriptional regulation in placental cells or is secondary to SARS-CoV-2-associated placental alterations. It remains possible that SARS-CoV-2 induced upregulation of toxic pHERVW ENV directly produces the tissue damage that we observed, similar to pHERVW ENV induced neuronal damage (van Horssen et al., 2016).

Further research and functional studies in *in vitro* models are required to establish both the order of events and a potential causal role for ENV. It also stands to reason that IFNβ is involved in these events, as high, local production acts against the integrity of the STB barrier (Ding et al., 2022).

## 5 Weakness of the study

A limitation of this study is the small number of SARS-COV-2 positive placentas with low Ct (*n* = 3) indicative of high viral load in placental tissue. These cases were included among SARS-CoV-2 positive placental samples in Figures 1–3, but were analyzed as a separate high-viral-load subgroup only in Figure 1C. Results from this specific subgroup should therefore be interpreted as exploratory. Another limitation is that exclusive recognition of pHERVW ENV cannot be fully confirmed in human tissue sections; therefore, the findings should be interpreted with appropriate caution.

## 6 Conclusion

We show that maternal COVID-19 disease is associated with a general downregulation of most placental TE elements, especially HERVW. We also demonstrate that pHERVW ENV protein is abundantly expressed in the STB and that its expression is dysregulated in placentas from COVID-19-affected pregnancies.

## Data Availability

The sequencing data supporting the conclusions of this article are available in the GEO repository under accession number GSE313868 (https://www.ncbi.nlm.nih.gov/geo/query/acc.cgi?acc=GSE313868).

## Footnotes

1
https://knowledge.illumina.com/library-preparation/general/library-preparation-general-reference_material-list/000003275

2
https://www.bioinformatics.babraham.ac.uk/projects/fastqc/
