# Correction: Alterations of placental HERV expression as a result of gestational COVID-19 disease

**DOI:** 10.3389/fmicb.2026.2018706

**Published:** 2026-10-21

**Authors:** Frontiers Production Office

**Affiliations:** Frontiers Media SA, Lausanne, Switzerland

**Keywords:** COVID-19, HERVW ENV, human endogenous retroviruses, placenta, pregnancy, placental damage, adverse pregnancy outcomes (APOs)

The author list and affiliation list were erroneously misaligned for authors A. Medel-Martínez, M. Bugatti, M. Polychronopoulou, M. Strunk, S. Macías-Redondo, A. Bazo, M. Garrido-Pontnou, K. Kitsou, M. Fabre, C. Paules, D. Oros, J. Pierquin, J. Brunel, B. Charvet, H. Perron, F. Facchetti, G. Magiorkinis, and J. Schoorlemmer.

The correct author list and full affiliation list appear below:

A. Medel-Martínez^1, 2, 3^, M. Bugatti^4†^, M. Polychronopoulou^5†^, M. Strunk^3^, S. Macías-Redondo^3‡^, A. Bazo^3^, M. Garrido-Pontnou^6^, K. Kitsou^5^, M. Fabre^1, 7, 8, 9^, C. Paules^1, 2, 9, 10, 11^, D. Oros^1, 7, 9, 10, 11^, J. Pierquin^12^, J. Brunel^12^, B. Charvet^12^, H. Perron^12^, F. Facchetti^4^, G. Magiorkinis^5^, and J. Schoorlemmer^3, 7, 10, 13*^

^1^Instituto de Investigación Sanitaria de Aragón (IIS Aragón), Zaragoza, Spain

^2^Maternal and Fetal Health Research Group, GIIS-106, IISA, Zaragoza, Spain

^3^Instituto Aragonés de Ciencias de la Salud (IACS), Centro de Investigación Biomédica de Aragón (CIBA), Zaragoza, Spain

^4^Pathology Unit, Department of Molecular and Translational Medicine, University of Brescia, Brescia, Italy

^5^Department of Preventive Medicine, Epidemiology and Medical Statistics, Medical School, National and Kapodistrian University of Athens, Athens, Greece

^6^Pathology Department, Hospital Universitari Vall d'Hebron, Barcelona, Spain

^7^Placental Pathophysiology & Fetal Programming Research Group, GIIS-028, IISA, Zaragoza, Spain

^8^Biochemistry Department, Hospital Clínico Universitario Lozano Blesa, Zaragoza, Spain

^9^Obstetrics Department, Hospital Clínico Universitario Lozano Blesa, Zaragoza, Spain

^10^Red de Salud Materno Infantil y del Desarrollo (SAMID), RETICS, Instituto de Salud Carlos III (ISCIII), Subdirección General de Evaluación y Fomento de la Investigación y Fondo Europeo de Desarrollo Regional (FEDER), Madrid, Spain

^11^University of Zaragoza, Zaragoza, Spain

^12^GeNeuro Innovation, Lyon, France

^13^ARAID Foundation, Zaragoza, Spain

^*^ **Correspondence:**

J. Schoorlemmer

jonas@araid.es

^†^These authors share second authorship

^‡^Present address: S. Macías-Redondo, Certest Biotec, Zaragoza, Spain

There were typographical errors, punctuation errors, and missing in-text citations in the published article.

A correction has been made to the **Section 1** (paragraph 1):

“...Kazazian, 2004. Among...”

A correction has been made to the **Section 1** (paragraph 2):

“...García-Montojo et al., 2018). HERVK consist of...”

“...its expression in villous and extravillous cytotrophoblast cells has been suggested to promote immune protection of the fetus during pregnancy (Kämmerer et al., 2011).”

A correction has been made to the **Section 1**, paragraph 3:

“On the other hand, elevated expression of HERVs has been associated with autoimmune disorders.”

A correction has been made to the **Section 2.5** heading:

“...HEK293T cells...”

A correction has been made to the **Section 4**, paragraph 2:

“...overall downregulation of HERVs...”

A correction has been made to *Section 3.1* paragraph 4:

“Since several of our assays (ERVWE1, HERVW ENV, and MSRV ENV) targeted closely related sequences derived from the HERVW family...”

There was a mistake in the caption of Figure 1 as published. The fold-change description contained a typographical error (“preCOVID19” instead of “preCOVID-19”) and the threshold key for four asterisks duplicated the three-asterisk threshold.

The corrected caption of Figure 1 appears below:

Analysis of retrotransposon expression in placental samples from women with COVID-19 during pregnancy using qPCR. Women who were qPCR-positive for SARS-CoV-2 (*n* = 32) were separated into two groups, either with a positive test in placenta (*n* = 14) or not (*n* = 18). The latter group was further divided according to the Ct value obtained (Ct < 30; *n* = 3; Ct > 30; *n* = 11). The presence of SARS-CoV-2 in placenta was measured by standard diagnostic qPCR (see M&M). Expression was compared to a group of preCOVID-19 placentas (*n* = 10) delivered by women with uneventful pregnancies. Box plots showing median expression and interquartile ranges (IQR) of placentas from **(A)** all SARS-CoV-2-infected pregnant women (*n* = 32) compared with preCOVID-19 uneventful controls (*n* = 10) and **(B)** women who were qPCR-positive for SARS-CoV-2 (*n* = 32) separated into two groups, either with a positive test in placenta (*n* = 14) or not (*n* = 18). The statistical significance of the differences defined by the *p*-value is indicated: *p* < 0.05 (^*^); *p* < 0.01 (^**^); *p* < 0.001 (^***^); *p* < 0.0001 (^****^). **(C)** The table shows the data recalculated as fold expression compared to the median of expression in the preCOVID-19 placentas. Fold change of controls (preCOVID-19 placentas) is set to 1.

There was a mistake in the caption of Figure 5 as published. The hyphenation of “preCOVID-19” was inconsistent. The corrected caption of Figure 5 appears below:

Reduced presence of pHERVW ENV protein after SARS-CoV-2 infection. Placental sections were co-stained with antibodies targeting SARS-CoV-2 nucleocapsid protein (NC), HCGβ, and pHERVW ENV protein. Staining was detected by multiplex tyramide signal amplification immunofluorescence (TSA-IF). Nuclei were stained with DAPI (blue). **(A)** Representative microscopy images are shown of stains carried out on sections from preCOVID-19 placentas (*n* = 3), placentas collected after pregnancies affected by COVID-19 disease, with either a SARS-CoV-2 positive test in placenta (*n* = 3) or not (*n* = 3). NC-staining was not homogeneous throughout sections, images with NC-positive cells have been selected (see squares). Note that even in selected areas, NC-positive and negative cells coexist. Images are acquired in single-channel mode using magnification 60 × . Scale bar: 100 μm. **(B)** Representative areas of the synctiotrophoblast cells positive for pHERVW ENV in each group. **(C)** Quantification of HCGβ positive cells per mm^2^. **(D)** Quantification of synctiotrophoblast cells positive for both HCGβ and pHERVW ENV per mm^2^. **(E)** Quantification of pHERVW ENV-expressing synctiotrophoblasts. The ratio between cells positive for pHERVW ENV and the number of synciotrophoblasts is expressed as a percentage. Five random fields per placental section were automatically selected and synctiotrophoblast cells were counted in single channel mode.

The original version of this article has been updated.

