# Supplementary material for: Alterations of placental HERV expression as a result of gestational COVID-19 disease
Source: Front Microbiol. 2026 Sep 11;17:1901506. doi: 10.3389/fmicb.2026.1901506 (PMC13612438; doi:10.3389/fmicb.2026.1901506)
Supplement: Supplementary file 1 [file Table_1.DOCX]

Supplementary material

**Figure S1. qPCR primer efficiency standard curves analysis.** A qPCR standard curve is graphically represented as a semi-log regression line plot of Ct value versus log of input or cDNA

**B**

**A**

| Placenta ID | RTase condition | *RPL13* | *RPL19* | *HERVW ENV* | *MSRV ENV* | *ERVWE1* | *ERVRD1* | *HERVK ENV* | *HERVK POL* | *HEMO* | *HERVH* |
| --- | --- | --- | --- | --- | --- | --- | --- | --- | --- | --- | --- |
| Sample 1 | **With RTase** | 18.471 | 26.851 | 18.219 | 23.755 | 19.732 | 21.695 | 23.623 | 23.842 | 23.295 | 24.297 |
|  | No RTase | 40.000 | 37.835 | 40.000 | 40.000 | 39.300 | 35.450 | 34.916 | 35.113 | 36.929 | 34.699 |
| Sample 2 | **With RTase** | 18.470 | 26.961 | 18.833 | 23.983 | 22.241 | 19.648 | 22.805 | 23.460 | 23.562 | 23.142 |
|  | No RTase | 30.605 | 34.487 | 31.934 | 39.167 | 36.063 | 33.525 | 31.968 | 31.989 | 40.000 | 31.680 |
| Sample 3 | **With RTase** | 19.127 | 28.724 | 20.818 | 24.471 | 23.200 | 21.921 | 24.830 | 24.996 | 24.854 | 27.363 |
|  | No RTase | 34.991 | 36.817 | 33.212 | 39.360 | 39.199 | 34.096 | 32.854 | 32.508 | 40.000 | 34.420 |
| Sample 4 | **With RTase** | 18.547 | 26.462 | 18.357 | 23.751 | 21.562 | 19.391 | 23.172 | 23.249 | 24.066 | 23.874 |
|  | No RTase | 34.998 | 39.324 | 34.558 | 40.000 | 34.623 | 34.979 | 33.003 | 32.965 | 40.000 | 33.587 |
| Sample 5 | **With RTase** | 18.386 | 27.521 | 23.427 | 23.424 | 25.113 | 24.425 | 22.168 | 23.435 | 27.671 | 21.535 |
|  | No RTase | 31.708 | 35.869 | 32.815 | 40.000 | 35.589 | 34.510 | 32.213 | 32.378 | 40.000 | 32.481 |
| Sample 6 | **With RTase** | 18.929 | 27.148 | 18.673 | 23.963 | 22.181 | 19.921 | 23.971 | 24.017 | 24.151 | 24.881 |
|  | No RTase | 31.900 | 35.258 | 33.271 | 40.000 | 40.000 | 36.151 | 32.095 | 33.909 | 35.419 | 35.567 |
| Sample 7 | **With RTase** | 19.216 | 27.089 | 19.317 | 24.671 | 23.778 | 20.943 | 23.204 | 23.869 | 25.083 | 22.532 |
|  | No RTase | 31.827 | 37.295 | 34.676 | 40.000 | 40.000 | 35.183 | 32.312 | 34.385 | 40.000 | 34.669 |

**Figure S2. Analysis of HERV amplification as a function of retrotranscriptase (RTase) activity**. **A)** RNA samples (n=7) were mock reverse transcribed with or without RTase. Resulting samples were used as a template to be amplified by qPCR. Amplification with or without previous RTase step was measured as Ct value, the difference was calculated (ΔCt value) and recalculated as fold change. The figure shows the fold change for each sample represented in logarithmic scale.  **B)** Paired Ct values in the same placental samples with and without RTase. Each placenta is shown as a paired two-row block: the RTase-positive reaction and its matched no-RTase control. places. Ct = 40.000 indicates no amplification within the assay cutoff.

**Figure S3**. **Comparison of PBMC and placental HERV expression levels.** PBMCs derived from healthy donors (n=8) and placentas derived from uneventful pregnancies (n=8) were analyzed by standard diagnostic qPCR.  The table shows the data as a fold change expression. Stars indicate levels of significance defined by the p-value.  p<0.05 (*); p<0.01 (**); p<0.001 (***); p<0.001 (****).

**Figure S4. Correlation Between SARS-CoV-2 load and HERV Expression in placentas with a SARS-CoV-2 positive test determined by qPCR (n = 14)**. Correlations between SARS-CoV-2 load and HERV expression were analyzed by using the Ct values extracted from previous RT-qPCR assays. Each dot represents a sample with expression data for both genes. The plots display linear regression lines, *P* values, and correlation coefficients (R2).

**Table S1.** **Baseline and clinical characteristics of the samples collected in HCULB (Zaragoza, Spain) and analyzed by qPCR.**

|  | | Uneventful pregnancies preCOVID-19  (n=10) | PE  preCOVID-19  (n=10) | Maternal COVID, SARS-CoV-2 negative placenta  (n=18) | Maternal COVID, SARS-CoV-2 positive placenta  (n=14) | p-value |
| --- | --- | --- | --- | --- | --- | --- |
| Maternal demographics | | | | | | |
| Maternal age, years, mean (SD) | | 31.18 (4.57) | 31.83 (5.84) | 31.48 (5.6) | 30.52 (6.57) | 0.93 |
| Maternal weight, kg, mean (SD) | | 59.30 (4.34) | 59.00 (7.10) | 68.00 (12.65) | 65.81 (10.43) | 0.05 |
| Maternal ethnicity, caucasian, n (%) | | 9 (90) | 3 (30) | 8 (44.44) | 6 (42.86) | **0.01** |
| Comorbidities | | | | | | |
| Obesity, (BMI>30), n (%) | | 0 (0) | 2 (20) | 5 (27.78) | 1 (7.14) | 0.47 |
| Labor and delivery | | | | | | |
| Parity, nulliparous, n (%) | | 5 (50) | 7 (70) | 9 (50) | 9 (64.29) | 0.65 |
| Vaginal delivery, n (%) | | 8 (80) | 5 (50) | 18 (100) | 10 (71.43) | 0.35 |
| Gestational age at birth, weeks, mean (SD) | | 39.9 (1.10) | 38.47 (0.95) | 39.25 (1.19) | 38.45 (1.76) | **0.03** |
| Pregnancy complications | | | | | | |
| Hypertensive disorders of pregnancy, n (%) | **HELLP syndrome** | 0 (0) | 0 (0) | 0 (0) | 1 (7.14) | **<0.001** |
|  | **Gestational HTA** | 0 (0) | 0 (0) | 4 (22.22) | 2 (14.28) |  |
|  | **Preeclampsia** | 0 (0) | 10 (100) | 1 (5.56) | 5 (35.71) |  |
| Preterm (<37 weeks gestation), n (%) | | 0 (0) | 1 (10) | 0 (0) | 2 (14.29) | 1 |
| Gestational diabetes, n (%) | | 0 (0) | 5 (5) | 1 (5.56) | 1 (7.14) | **0.03** |
| FGR, n (%) | | 0 (0) | 2 (20) | 2 (11.11) | 1 (7.14) | 0.47 |
| COVID-19 diagnostic criteria | | | | | | |
| COVID-19 symptoms, n (%)  Asymptomatic  Mild  Severe | |  |  | 10 (55.56)  8 (44.44)  0 (0) | 7 (50)  6 (42.86)  1 (7.14) | 0.51 |
| Interval from maternal SARS-CoV-2 infection to delivery, n (%)  <3 months  ≥3 months | |  |  | 13 (72)  5 (28) | 7 (50)  7 (50) | 0.27 |
| Fetal demographics | | | | | | |
| Birth weight, g, mean (SD) | | 3476.00 (256.74) | 2740.00 (383.78) | 3136.91 (457.24) | 3035.8 (560.95) | **5.31e-07** |
| Fetal gender, male, n (%) | | 6 (60) | 3 (30) | 11 (61.11) | 6 (42.86) | 0.36 |

Data are expressed as mean ± standard deviation, or number of cases n (%). BMI, body mass index; HDP, hypertensive disorder of pregnancy (chronic hypertension with superimposed preeclampsia, gestational hypertension (HTA), preeclampsia [1]; FGR, fetal growth restriction. COVID-19-specific variables were compared between groups with maternal COVID-19. Significance: p < 0.05.

**Table S2, Portrait orientation, individual file**

**Table S3. Baseline characteristics of the pregnancies included in the Zaragoza group used for HERV RNA quantification by RNA-seq.**

|  | | preCOVID-19 (n=10) | SARS– placenta negative (n=24) | SARS+ placenta positive (n=16) | p-value |
| --- | --- | --- | --- | --- | --- |
| Maternal demographics | | | | | |
| Maternal age, years, mean (SD) | | 31.18 (4.57) | 31.20 (5.99) | 31.92 (6.61) | 0.92 |
| Maternal weight, kg, mean (SD) | | 59.30  (4.34) | 68.20 (12.74) | 67.13 (10.19) | 0.09 |
| Maternal ethnicity, caucasian, n () | | 9 (90) | 8 (33.33) | 13 (81.25) | **0.001** |
| Comorbidities | | | | | |
| Obesity (BMI >30), n () | | 0 (0) | 6 (25) | 1 (6.25) | 0.09 |
| Labor and delivery | | | | | |
| Nulliparous, n (%) | | 5 (50) | 11 (45.83) | 10 (62.50) | 0.58 |
| Vaginal delivery, n (%) | | 8 (80) | 24 (100) | 11 (68.75) | **0.017** |
| Gestational age at birth, weeks, mean (SD) | | 39.9 (1.10) | 38.29 (3.97) | 38.62 (1.76) | 0.36 |
| Pregnancy complications | | | | | |
| Hypertensive disorders of pregnancy, n (%) | **HELLP syndrome, n (%)** | 0 (0) | 0 (0) | 1 (6.25) | **<0.001** |
|  | **Gestational HTA, n (%)** | 0 (0) | 1 (4.17) | 2 (12.50) |  |
|  | **Preeclampsia, n (%)** | 0 (0) | 1 (4.17) | 6 (37.50) |  |
| Preterm <37 weeks, n (%) | | 0 (0) | 4 (16.67) | 2 (12.50) | 0.39 |
| Gestational diabetes, n (%) | | 0 (0) | 3 (12.5) | 1 (6.25) | 0.45 |
| FGR, n (%) | | 0 (0) | 1 (4.17) | 1 (6.25) | 0.73 |
| COVID-19 diagnostic criteria | | | | | |
| Asymptomatic | | — | 11 (45.83) | 8 (50) | 0.95 |
| Mild | | — | 11 (45.83) | 7 (43.75) |  |
| Severe | | — | 2 (8.33) | 1 (6.25) |  |
| <3 months to delivery | | — | 11 (45.83) | 9 (56.25) | 0.52 |
| ≥3 months to delivery | | — | 13 (54.17) | 7 (43.75) |  |
| Fetal demographics | | | | | |
| Birth weight, g, mean (SD) | | 3476  (257) | 3176  (724) | 3079.7 (529.6) | 0.26 |
| Fetal gender, male, n (%) | | 6 (60) | 14 (58.33) | 7 (43.75) | 0.60 |

Data are expressed as mean ± standard deviation, or number of cases n (%). BMI, body mass index; HDP, hypertensive disorder of pregnancy (chronic hypertension with superimposed preeclampsia, gestational hypertension (HTA), preeclampsia [87]; FGR, fetal growth restriction. COVID-19-specific variables were compared between groups with maternal COVID-19. Significance: p < 0.05.

**Table S4.** **List of primers and TaqMan Assays used for cDNA amplification.**

| **Target** | **Oligonucleotide** | **Source** | **Sequence** | **Reference** |
| --- | --- | --- | --- | --- |
| Syncytin-1  (*ERVWE1*) | Forward Primer | IDT | 5’-ATGGAGCCCAAGATGCAG-3’ | Ruebner et al. (2013) [2] |
|  | Reverse Primer | IDT | 5’AGATCGTGGGCTAGCAG-3’ |  |
| Syncytin-2  (*ERVFRD1*) | Forward Primer | IDT | 5’-GCTGTCCCTGGTGTTTCAGT-3’ | Vargas et al. (2009) [3] |
|  | Reverse Primer | IDT | 5’-CCTTCACTAGCAGCCTACCG-3’ |  |
| *HERVW ENV* | Forward Primer | IDT | 5’-GTATGTCTGATGGGGGTGGAG-3’ | Balestrieri et al. (2021) [4] |
|  | Reverse Primer | IDT | 5’-CTAGTCCTTTGTAGGGGCTAGAG-3’ |  |
| *MSRV ENV* | Forward Primer | IDT | 5’-CTTCCAGAATTGAAGCTGTAAAGC-3’ | Mameli et al. (2009) [5] |
|  | Reverse Primer | IDT | 5’-GGGTTGTGCAGTTGAGATTTCC-3’ |  |
|  | Probe: FAM- TAMRA | IDT | 5’-TTCTTCAAATGGAGCCCCAGATGCAG-3’ |  |
| *HERVK ENV* | Forward Primer | Sigma | 5’-CTGAGGCAATTGCAGGAGTT-3’ | Li et al. (2015) [6] |
|  | Reverse Primer | Sigma | 5’-GCTGTCTCTTCGGAGCTGTT-3’ |  |
| *HERVK POL* | Forward Primer | Sigma | 5’-TCACATGGAAACAGGCAAAA-3’ | Li et al. (2015) [6] |
|  | Reverse Primer | Sigma | 5’-AGGTACATGCGTGACATCCA-3' |  |
| *LINE-1* | Forward Primer | IDT | 5’-GAATGATTTTGACGAGCTGAGAGAA-3’ | Muñoz-Lopez, Jose L et al. (2012) [7] |
|  | Reverse Primer | IDT | 5’-GTCCTCCCGTAGCTCAGAGTAATT-3’ |  |
| *HEMO* | Forward Primer | IDT | 5′-ACTATGGGCTCCCTTTCAAACT-3’ | Heidmann et al. (2017) [8] |
|  | Reverse Primer | IDT | 5′-CATAGGAGGAAGTAGAGTGATT-3’ |  |
| *HERVH POL* | Forward Primer | IDT | 5’-TGGACTGTGCTGCCGCAA-3’ | Mareschi K. et al. (2019) [9] |
|  | Reverse Primer | IDT | 5’GAAGSTCATCAATATCCTCATTGAATAAGGTGAGA-3’ |  |
| *RPL13* | Forward Primer | IDT | 5’-AGCCTACAAGAAAGTTTGCCTAT-3’ | Rydbirk et al. (2016) [10] |
|  | Reverse Primer | IDT | 5’-TCTTCTTCCGGTAGTGGATCTTGGC-3’ |  |
| *RPL19* | Forward Primer | IDT | Hs.PT.58.20491224 |  |
|  | Reverse Primer | IDT |  |  |
|  | Probe: | IDT |  |  |
| *SDHA* | Forward Primer | IDT | Hs.PT.58.40170798 |  |
|  | Reverse Primer | IDT |  |  |
|  | Probe: | IDT |  |  |

**Table S5. List of reference sequences used for mapping of sequenced amplified products.**

|  | **Reference sequence** |
| --- | --- |
| *ERVWE1* | NC_000007.14:c92477946-92468380 ERVW-1 [organism=Homo sapiens] [GeneID=30816] |
| *ERVFRD1* | NC_000006.12:c11111725-11102489 ERVFRD-1 [organism=Homo sapiens] [GeneID=405754] |
| *HERVW ENV* consensus | Consensus sequence of the *ENV* gene of the HERVW family. Hervé Perron by personal communication [11] |
| *MSRV ENV* | cDNA clone from MS cell culture virion RNA encoding 542 amino acids (Gen- Bank no. AF331500.1) Mameli et al. (2009) [5] |
| *HERVK ENV* | AY037928.1 Human endogenous retrovirus K113 complete genome |
| *HERVK POL* | AY037928.1 Human endogenous retrovirus K113 complete genome |
| *LINE-1* | NM_019079.5 |
| *HEMO* | NM_024534.6 Homo sapiens endogenous retrovirus group MER34 member 1, envelope (ERVMER34-1) |
| *HERVH* | AJ289710.2 Human endogenous retrovirus H HERV-H/env60 proviral copy, clone 734E12 |
| *RPL13A* | NM_001270491.2 RPL13 [organism=Homo sapiens] [GeneID=6137] |
| *RPL19* | NM_000981.4 RPL19 [organism=Homo sapiens] [GeneID=6143] [transcript=1]; NM_001330200.1 RPL19 [organism=Homo sapiens] [GeneID=6143] [transcript=2] |
| *SDHA* | NM_004168.4 SDHA [organism=Homo sapiens] |

Reference sequences were generated for *HERVK ENV*, *HERVK POL, MSRV ENV* and the *HERVW ENV* consensus sequence, as these were not included in the annotated reference genome. The sequences were prepared using Bowtie2-build and the trimmed FASTQ files.

**Table S6. Validation of sequenced qPCR amplified products.**

| **Amplicon** | **Reference** | **Reads** | **% mapped** |
| --- | --- | --- | --- |
| **ERVWE1** | ***ERVWE1*** | 975765 | 100 |
| **ERVFRD1** | ***ERVFRD1*** | 4640530 | 100 |
| **HERVW ENV** | ***HERVW ENV***  **consensus** | 1152795 | 96.4 |
| **MSRV ENV** | ***MSRV ENV*** | 884665 | 97.8 |
| **HERVK ENV** | ***HERVK ENV*** | 1355 | 100 |
| **HERVK POL** | ***HERVK POL*** | 327350 | 100 |
| **LINE-1** | ***LINE-1*** | 280232 | 100 |
| **HEMO** | ***HEMO*** | 39315 | 97.4 |
| **HERVH** | ***HERVH*** | 3199 | 97 |
| **RPL13A** | ***RPL13A*** | 34448 | 100 |
| **RPL19** | ***RPL19*** | 213395 | 100 |
| **SDHA** | ***SDHA*** | 360646 | 96 |

Amplified products from a pool of samples (n=8) obtained through qPCR were sequenced to confirm that they corresponded to the target transcripts (see Tables S4 and S5). The table presents the number of sequencing reads obtained per gene and the percentage of reads successfully mapped to the reference sequences. Mapping percentages indicate high specificity, with most genes showing near-complete alignment to their reference sequences. The reads were mapped to the reference using Bowtie 2 program version 2.5.2 using “very-sensitive-local” and “k 1” options.

**Table S7. Analysis of cross-mapping.**

|  | | **% reads mapped** | | | |
| --- | --- | --- | --- | --- | --- |
|  |  | **Reference sequences** | | | |
|  |  | ***ERVWE1*** | ***ERVFRD1*** | ***HERVW* consensus** | ***MSRV ENV*** |
| **Assays** | **ERVWE1** | 100.00 | 0.00 | 0.29 | 1.44 |
|  | **ERVFRD1** | 0.00 | 100.00 | 0.00 | 0.00 |
|  | **HERVW ENV** | 0.23 | 0.00 | 100 | 100 |
|  | **MSRV ENV** | 1.46 | 0.00 | 100 | 100 |

Sequences obtained by NGS were aligned to the reference sequences indicated in Table S5. Values shown in this table indicate the percentage of sequenced reads aligned to a reference sequence.

**Table S8**. **Summary of statistical analyses.** P values corresponding to all comparisons of median gene expression presented in Figure 1C, including preCOVID-19 uneventful pregnancies versus SARS-CoV-2–infected pregnancies, and subgroup analyses according to placental SARS-CoV-2 detection by standard diagnostic qPCR (positive vs negative), for all analyzed HERVs.

|  | **preCOVID-19 Uneventful vs COVID mother** | **preCOVID-19 Uneventful vs neg placenta** | **preCOVID-19 Uneventful vs pos placenta** | **preCOVID-19 Uneventful vs Ct<30** | **preCOVID-19 Uneventful vs Ct>30** | **Neg vs pos placenta** | **Ct<30 vs Ct>30** |
| --- | --- | --- | --- | --- | --- | --- | --- |
| ***ERVWE1*** | **0.0643** | 0.1458 | **0.0602** | **0.0280** | 0.1597 | 0.5360 | 0.3681 |
| ***ERVFRD1*** | **0.0008** | **0.0406** | **0.0003** | **0.0769** | **0.0004** | **0.0485** | 0.5549 |
| ***HERVW ENV*** | 0.3136 | 0.555 | 0.2125 | **0.0070** | 0.6539 | 0.3772 | 0.4560 |
| ***MSRV ENV*** | **0.0558** | **0.0716** | **0.0118** | **0.0490** | **0.0349** | 0.1773 | 0.2253 |
| ***HERVK ENV*** | 0.4575 | 0.7956 | 0.1862 | 0.287 | 0.3787 | 0.2581 | 0.6593 |
| ***HERVK POL*** | **0.0197** | 0.2725 | **0.0003** | 0.4685 | **0.0020** | **0.0161** | 0.2867 |
| ***LINE-1*** | 0.4060 | 0.4937 | 0.8543 | 0.3706 | 0.9856 | 0.9254 | 0.5549 |
| ***HEMO*** | **0.009** | **0.0229** | **0.0029** | **0.0069** | **0.0115** | 0.1716 | 0.5549 |
| ***HERVH POL*** | 0.585 | 0.465 | 0.8859 | 0.5734 | 0.9725 | 0.1509 | 0.8846 |

**Table S9. Summary of the study design.** Samples analyzed, study groups, collection place and the technique used to assess HERVs in each sample are reported.

| Group | Sampling site | Maternal SARS-CoV-2 status | Placental SARS-CoV-2 status | RNA-seq, | qPCR | IHC / TSA-IF |
| --- | --- | --- | --- | --- | --- | --- |
| preCOVID-19 Uneventful pregnancies | Zaragoza (Spain) | — | Negative | n=10 | n=10 | — |
| preCOVID-19 Preeclampsia | Barcelona (Spain) | — | Negative | n=10 | n=10 | — |
| Gestational COVID-19, SARS– placenta | Zaragoza (Spain) | Positive | Negative | n=13 | n=15 | — |
| Gestational COVID-19 SARS+ placenta | Zaragoza (Spain) | Positive | Positive | n=17 | n=14 | — |
| preCOVID-19 | Brescia (Italy) | Negative | Negative | — | — | n=3 |
| Gestational COVID-19 SARS– placenta | Brescia (Italy) | Positive | Negative | — | — | n=3 |
| Gestational COVID-19 SARS+ placenta | Brescia (Italy) | Positive | Positive | — | — | n=3 |

RNA-seq and qPCR were performed on **the same cohort of samples**, with **overlapping subsets** depending on tissue availability and RNA quality. Maternal SARS-CoV-2 status was determined by RTqPCR from nasal swab samples, while placental SARS-CoV-2 status was determined by RTqPCR analysis of placental parenchyma tissue. IHC, immunohistochemistry; IF, immunofluorescence.

**Table S10**. **Comparison of HERV differential expression between placentas from gestational COVID-19 pregnancies (n=40) and preCOVID-19 controls (n=10), and between COVID-19 cases stratified by the presence of the SARS-CoV-2 genome in placental tissue.**

|  | Comparison between placental samples from pregnant women with gestational COVID-19 (n=40) and preCOVID-19 samples from uneventful pregnancies (n=10) | | Comparison between SARS-CoV-2(+) placenta (n=16) and SARS-CoV-2 (-) placenta (n=24) | |
| --- | --- | --- | --- | --- |
| HERV group | **Fold change** | ***p-value*** | **Fold change** | ***p-value*** |
| *HERVK HML-1* | 1.04 | 0.816 | 0.90 | 0.407 |
| *HERVK HML-2* | 1.05 | 0.800 | 0.93 | 0.343 |
| *HERVK HML-2 Solo LTRs* | 1.03 | 0.333 | 1.05 | 0.349 |
| *HERVK HML-3* | 0.88 | 0.119 | 0.87 | 0.100 |
| *HERVK HML-4* | 0.88 | 0.099 | 0.90 | 0.245 |
| *HERVK HML-5* | 0.96 | 0.545 | 0.88 | 0.184 |
| *HERVK HML-6* | 0.91 | 0.107 | *0.61* | **0.022** |
| *HERVW* | 0.86 | **0.019** | 0.71 | **0.019** |
| *HERVFRD* | 0.92 | 0.073 | 0.75 | **0.020** |
| *ERVWE1* | 0.85 | **0.014** | 0.70 | **0.020** |
| *HERVH* | 0.95 | 0.308 | 0.85 | 0.065 |
| *HERVI* | 0.85 | 0.080 | 0.80 | **0.041** |
| *HERVL* | 0.89 | 0.080 | 0.94 | 0.136 |
| *HERVE* | 0.91 | 0.096 | 0.85 | 0.097 |
| *HERV9* | 0.90 | 0.283 | 0.78 | **0.012** |

Normalized read counts from each HERV group were calculated, the corresponding fold changes between the patient groups indicated were calculated. Resulting p-values were determined based on the log10-transformed normalized read counts.

**Table S11.** **Analysis of retrotransposon expression in placental samples in** **women diagnosed with PE (n=10) and positive placentas from women with COVID-19 disease during pregnancy, either suffering from HDP (n=9) or not (n=5).**

|  | **Uneventful pregnancies preCOVID-19** | **PE preCOVID-19** | **Maternal, COVID, SARS-CoV-2 positive placenta** | **Maternal, COVID, SARS positive placenta, no HDP** | **Maternal COVID, SARS positive placenta, HDP** |
| --- | --- | --- | --- | --- | --- |
|  | **n=10** | **n=10** | **n=14** |  |  |
|  |  |  |  | **n=5** | **n=9** |
| ***ERVWE1*** | 1 | 0.59 | 0.63 | 0.06 | 0.85 |
| ***ERVFRD1*** | 1 | 0.47 | 0.65 | 0.25 | 0.7 |
| ***HERVW ENV*** | 1 | 0.63 | 0.55 | 0.06 | 1.07 |
| ***MSRV ENV*** | 1 | 0.67 | 0.66 | 0.59 | 0.77 |
| ***HERVK ENV*** | 1 | 1.03 | 1.06 | 1.5 | 1.03 |
| ***HERVK POL*** | 1 | 0.48 | 0.58 | 0.49 | 0.67 |
| ***LINE-1*** | 1 | 1.13 | 0.93 | 0.97 | 0.75 |
| ***HEMO*** | 1 | 0.4 | 0.45 | 0.11 | 0.59 |
| ***HERVH POL*** | 1 | 1.44 | 1.15 | 5.24 | 0.78 |

The presence of SARS-CoV-2 in placenta was measured by standard diagnostic qPCR. The table shows the data as a fold change expression compared to the median of expression level in a group of preCOVID-19 placentas (n=10) delivered by women with uneventful pregnancies. PE, preeclampsia; HDP, hypertensive disorders of pregnancy.

1. Brown MA, Magee LA, Kenny LC, Karumanchi SA, McCarthy FP, Saito S, et al. Hypertensive Disorders of Pregnancy: ISSHP Classification, Diagnosis, and Management Recommendations for International Practice. Hypertension. 2018;72:24–43. https://doi.org/10.1161/HYPERTENSIONAHA.117.10803

2. Ruebner M, Strissel PL, Ekici AB, Stiegler E, Dammer U, Goecke TW, et al. Reduced Syncytin-1 Expression Levels in Placental Syndromes Correlates with Epigenetic Hypermethylation of the ERVW-1 Promoter Region. Oudejans C, editor. PLoS ONE. 2013;8:e56145. https://doi.org/10.1371/journal.pone.0056145

3. Vargas A, Moreau J, Landry S, LeBellego F, Toufaily C, Rassart E, et al. Syncytin-2 plays an important role in the fusion of human trophoblast cells. J Mol Biol. 2009;392:301–18. https://doi.org/10.1016/j.jmb.2009.07.025

4. Balestrieri E, Minutolo A, Petrone V, Fanelli M, Iannetta M, Malagnino V, et al. Evidence of the pathogenic HERV-W envelope expression in T lymphocytes in association with the respiratory outcome of COVID-19 patients. EBioMedicine. 2021;66:103341. https://doi.org/10.1016/j.ebiom.2021.103341

5. Mameli G, Poddighe L, Astone V, Delogu G, Arru G, Sotgiu S, et al. Novel reliable real-time PCR for differential detection of MSRVenv and syncytin-1 in RNA and DNA from patients with multiple sclerosis. J Virol Methods. 2009;161:98–106. https://doi.org/10.1016/j.jviromet.2009.05.024

6. Li W, Lee M-H, Henderson L, Tyagi R, Bachani M, Steiner J, et al. Human endogenous retrovirus-K contributes to motor neuron disease. Sci Transl Med. 2015;7:307ra153. https://doi.org/10.1126/scitranslmed.aac8201

7. Muñoz-Lopez M, Garcia-Cañadas M, Macia A, Morell S, Garcia-Perez JL. Analysis of LINE-1 expression in human pluripotent cells. Methods Mol Biol. 2012;873:113–25. https://doi.org/10.1007/978-1-61779-794-1_7

8. Heidmann O, Béguin A, Paternina J, Berthier R, Deloger M, Bawa O, et al. HEMO, an ancestral endogenous retroviral envelope protein shed in the blood of pregnant women and expressed in pluripotent stem cells and tumors. Proc Natl Acad Sci USA [Internet]. 2017 [cited 2025 Feb 27];114. https://doi.org/10.1073/pnas.1702204114

9. Mareschi K, Montanari P, Rassu M, Galliano I, Daprà V, Adamini A, et al. Human Endogenous Retrovirus-H and K Expression in Human Mesenchymal Stem Cells as Potential Markers of Stemness. Intervirology. 2019;62:9–14. https://doi.org/10.1159/000499185

10. Rydbirk R, Folke J, Winge K, Aznar S, Pakkenberg B, Brudek T. Assessment of brain reference genes for RT-qPCR studies in neurodegenerative diseases. Sci Rep. 2016;6:37116. https://doi.org/10.1038/srep37116

11. Brunel J, Paganini J, Galloux M, Charvet B, Perron H. HERV-W ENV transcription in B cells predicting symptomatic COVID-19 and risk for long COVID can express a full-length protein despite stop codon in mRNA from chromosome X via a ribosome readthrough. Microbes Infect. 2024;105431. https://doi.org/10.1016/j.micinf.2024.105431
