# Supplementary material for: Alterations of placental HERV expression as a result of gestational COVID-19 disease
Source: Front Microbiol. 2026 Sep 11;17:1901506. doi: 10.3389/fmicb.2026.1901506 (PMC13612438; doi:10.3389/fmicb.2026.1901506)
Supplement: Supplementary file 2 [file Table_2.DOCX]

| Number | CTRL#1 | CTRL#2 | CTRL#3 | COVID positive mother#1 | COVID positive mother#2 | COVID positive mother#3 | COVID positive mother#4 | COVID positive mother#5 | COVID positive mother#6 |
| --- | --- | --- | --- | --- | --- | --- | --- | --- | --- |
| Group | preCOVID-19 | | | SARS-CoV-2 Negative Placenta | | | SARS-CoV-2 Positive Placenta | | |
| Mother Age, years | 32 | 19 | 20 | 40 | 24 | 28 | 29 | 31 | 29 |
| Mother Condition | Diabetes | Preeclampsia | Hypertension | COVID-19 Suspected, Severe IUGR, Preeclampsia | COVID-19 Positive, Premature rupture of membranes (PROM) | COVID-19 Positive | SARS-CoV-2 positive, fever (38°C), idiopathic thrombocytopenia, COVID-19 pneumonia (3/18 score) | SARS-CoV-2 positive, severe pneumonia, CID | SARS-CoV-2 positive (asymptomatic), preeclampsia, HELLP syndrome |
| Gestational Age (weeks) | 39 | 39 | 37+5 | 32+6 | 40+4 | 39+2 | 37+5 | 32+1 | 26+3 |
| Delivery Mode | Induced vaginal delivery | Spontaneous vaginal delivery | Spontaneous vaginal delivery | Elective cesarean due to IUGR and venous duct alterations | Emergency cesarean due to vacuum extraction failure | Induced vaginal delivery | Induced vaginal delivery due to fever and worsening thrombocytopenia, with supportive ventilation | Cesarean due to severe COVID-19 pneumonia and CID | Cesarean due to preeclampsia and HELLP syndrome |

**Table S2. Baseline and clinical characteristics of the samples collected in BSCH (Brescia, Italy) and analyzed by immunofluorescence.**
